# BODIPY‐Functionalized Natural Polymer Coatings for Multimodal Therapy of Drug‐Resistant Bacterial Infection

**DOI:** 10.1002/advs.202300328

**Published:** 2023-03-19

**Authors:** Lujiao Zhang, Chenyan Hu, Meizhou Sun, Xiaokang Ding, Hong‐Bo Cheng, Shun Duan, Fu‐Jian Xu

**Affiliations:** ^1^ State Key Laboratory of Chemical Resource Engineering State Key Laboratory of Organic‐Inorganic Composites Key Lab of Biomedical Materials of Natural Macromolecules (Beijing University of Chemical Technology) Ministry of Education Beijing Laboratory of Biomedical Materials Beijing 100029 China

**Keywords:** antibacterial, coating, photoacoustic imaging, photodynamic therapy, photothermal effect

## Abstract

The fact that multidrug resistance (MDR) could induce medical device‐related infections, along with the invalidation of traditional antibiotics has become an intractable global medical issue. Therefore, there is a pressing need for innovative strategies of antibacterial functionalization of medical devices. For this purpose, a multimodal antibacterial coating that combines photothermal and photodynamic therapies (PTT/PDT) is developed here based on novel heavy atom‐free photosensitizer compound, BDP‐6 (a kind of boron‐dipyrromethene). The photothermal conversion efficiency of BDP‐6 is of 55.9%, which could improve biocompatibility during PTT/PDT process by reducing the exciting light power density. Furthermore, BDP‐6, together with oxidized hyaluronic acid, is crosslinked with a natural polymer, gelatin, to fabricate a uniform coating (denoted as polyurethane (PU)‐GHB) on the surface of polyurethane. PU‐GHB has excellent synergistic in vitro PTT/PDT antibacterial performance against both susceptible bacteria and MDR bacteria. The antibacterial mechanisms are revealed as that hyperthermia could reduce the bacterial activity and enhance the permeability of inner membrane to reactive oxygen species by disturbing cell membrane. Meanwhile, in an infected abdominal wall hernia model, the notable anti‐infection performance, good in vivo compatibility, and photoacoustic imaging property of PU‐GHB are verified. A promising strategy of developing multifunctional antibacterial coatings on implanted medical devices is provided here.

## Graphical abstract

Multimodal photodynamic/photothermal antibacterial coatings with photoacoustic imaging property are fabricated based on natural polymers for therapy of drug‐resistant bacterial infection.

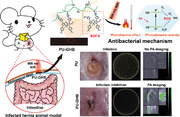

## 1 Introduction

Clinically, medical device‐induced bacterial infections are mainly caused by the bacteria that adhere and adapt to the substrate surface.^[^ ^1^ ^]^ In addition, the escalating tide of multidrug resistance (MDR) in bacterial infections has become an intractable medical issue globally, which introduces a tremendous economic burden and threatens human health seriously.^[^ ^2^, ^3^ ^]^ MDR bacteria have caused 5.3 million deaths and a third of patients in intensive care unit suffered from MDR bacterial infections each year.^[^ ^4^ ^]^ In a pessimistic scenario, MDR bacterial infection will become a main cause of death in 2050.^[^ ^5^ ^]^ The MDR bacteria, such as methicillin‐resistant *Staphylococcus aureus* (MRSA), represented a devastating clinical challenge.^[^ ^6^, ^7^, ^8^ ^]^ The latest data from World Health Organization shows that the average mortality associated with MRSA infections was ≈64% higher than that of susceptible *S. aureus*‐induced infections, while the recurrence of MRSA ranged from 18% to 43%.^[^ ^9^ ^]^ Therefore, to prevent medical device‐induced MDR bacterial infections, there is an urgent need for novel antibacterial coatings which can both treat MDR infections and prevent further resistance development by outsmarting the bacterial evolutionary mechanisms.

Tremendous efforts have been made to develop effective antibacterial strategies to solve the challenge of MDR bacteria, such as silver nanoparticles,^[^ ^10^, ^11^, ^12^ ^]^ metal–organic frameworks (MOFs),^[^ ^13^, ^14^ ^]^ metal oxides,^[^ ^15^, ^16^, ^17^ ^]^ antimicrobial peptides,^[^ ^18^, ^19^ ^]^ teixobactin,^[^ ^20^, ^21^ ^]^ and other materials.^[^ ^22^, ^23^, ^24^ ^]^ These antibacterial agents can achieve broad‐spectrum antibacterial activity via physical or chemical approaches. Nevertheless, they still have some disadvantages, such as high cost (e.g., noble metals), instability (e.g., MOFs), toxicity (e.g., cationic materials), and drug resistance (e.g., antibiotics). In addition, it takes at least 10 years for a new antibacterial drug before being used in clinical practice while it only takes the pathogen less than two weeks to develop MDR.^[^ ^25^ ^]^ We are still relying on traditional antibiotics^[^ ^26^, ^27^ ^]^ and it is necessary to find innovative strategies to overcome the problem of MDR bacterial infections.

With the emergence of the MDR bacterial infection, photothermal therapy (PTT) has been applied as an emerging therapeutic mode, which can inhibit bacterial infection without inducing drug resistance.^[^ ^28^ ^]^ PTT utilizes photothermal agents to kill MDR bacteria by converting the energy of near‐infrared (NIR) light into localized high temperature.^[^ ^29^, ^30^, ^31^ ^]^ The hyperthermia will not induce the development of MDR while killing bacteria by irreversible oxidation of the phospholipid layer of bacterial cell membrane, destruction of the structure of cell membrane, and protein denaturation.^[^ ^32^ ^]^ However, PTT treatment alone may result in an uneven distribution of thermal energy on the target tissue and may lead to recurrence of bacterial infection.^[^ ^33^ ^]^ In addition, PTT still has biocompatibility concerns because of overheat.^[^ ^34^ ^]^ Photodynamic therapy (PDT) is another light‐induced antibacterial strategy without producing drug resistance, which can combine photosensitizers and oxygen to attack bacteria by the reactive oxygen species (ROS) produced under appropriate excitation light.^[^ ^35^ ^]^ Compared with PTT, PDT is a less invasive technique with less damage to healthy tissues and cells around the infected site. It is considered to overcome the heat shock proteins (HSPs)‐mediated thermotolerance toward PTT.^[^ ^36^ ^]^ However, the efficacy of PDT is limited by the infection site and short life of ^1^O_2_.^[^ ^37^ ^]^ In view of the above‐mentioned problems, a combined strategy of PTT/PDT could be a better choice because the antibacterial activity of PDT could reduce the effective temperature of PTT.^[^ ^38^, ^39^ ^]^ The hyperthermia could reduce the cell viability and even promote the production of ROS by disturbing the structure of cell membrane, which could further aggravated the damage of the membrane structure, leading to leakage of intracellular contents.^[^ ^39^ ^]^ Conventional organic molecules and heavy metal atom‐containing compounds, which are, nevertheless, often limited by their photobleaching and low quantum yield could be utilized for generating singlet oxygen.^[^ ^40^, ^41^ ^]^ For the inorganic carbon‐ and silicon‐based nanomaterials, the lack of long wavelength absorption and poor biodegradability restricted their broad PDT studies in vivo.^[^ ^42^, ^43^ ^]^

In our previous work, we used a heavy‐atom‐free boron‐dipyrromethene (BODIPY) dye derivative with nanoparticulate form, BDP‐4, to describe a supramolecular host‐guest activation strategy.^[^ ^44^ ^]^ The BDP‐4 showed long‐wavelength absorption, high photothermal conversion efficiency (PCE), and high photostability, which provided a dual‐modal PTT/PDT therapeutic nanoplatform against tumor. Therefore, the synergetic PTT/PDT activities of BDP‐4 might kill MDR bacteria. However, there were still some shortcomings of BDP‐4 for antibacterial application against medical device‐induced infections. For example, high power densities (2 W cm^−2^ in vivo) might cause an uneven distribution of heat and phototoxicity. In addition, BDP‐4 was difficult to be integrated covalently into surface coatings of medical devices, because there were no active functional groups in the molecular structure of BDP‐4. Thus, the molecular structure of BDP‐4 needs to be improved to get higher PCE to reduce the NIR power densities in the therapeutic process, and to possess higher reactivity to form coatings by chemical reactions. What is more, a facile strategy to construct BODIPY‐based antibacterial coating on surfaces of medical devices is also challenging.

**Scheme 1:**
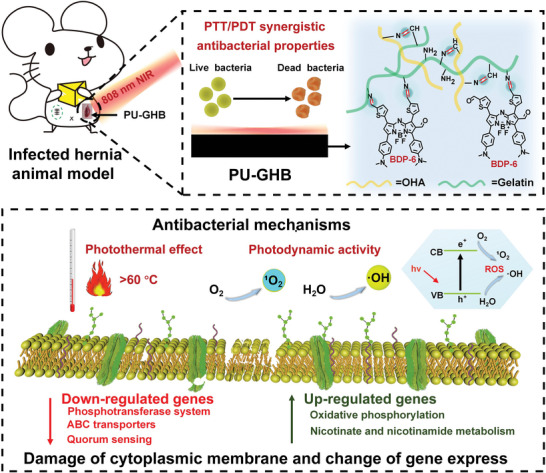
Schematic illustration. Schematic illustration of the properties and acting mechanisms of PU‐GHB.

In this work, we synthesized a novel BODIPY, BDP‐6, which had synergistic PTT/PDT effects and reactive functional groups (aldehyde groups) for fabricating coatings. Then, we used BDP‐6 and two kind of natural polymers, hyaluronic acid (HA) and gelatin, to construct the antibacterial coating on polyurethane (PU) hernia meshes, which was a typical implanted medical device (**Scheme** **1**). The mesh should have biocompatibility and the phenomena of deadhesion to avoid adherence to viscera.^[^ ^45^ ^]^ Oxidized HA (OHA) with multiple aldehyde groups has good biocompatibility and hydrophilic performances,^[^ ^46^, ^47^ ^]^ which can effectively prevent bacterial adhesion on the surface and the occurrence of tissue adhesion. Amino‐rich gelatin with good film‐forming property can regulate the thickness of surface coating and the loading amount of antibacterial agents.^[^ ^48^, ^49^ ^]^ The film forming solution was prepared by the rational combination of BDP‐6, OHA, and gelatin. Then, the coating (PU‐GHB) was modified on PU surface by a volatilization film‐forming method. PU‐GHB coatings were crosslinked by Schiff base reaction among the amino groups of gelatin and the aldehyde groups of BDP‐6 and OHA. The photothermal performances, antibacterial properties, and antibacterial mechanisms of PU‐GHB were verified in vitro, and the in vivo anti‐infection performance was evaluated by an MDR bacterial infected abdominal wall hernia model. Moreover, the in vivo photoacoustic (PA) imaging property of BDP‐6 was evaluated. This work could provide a realizable strategy to fabricate effective PTT/PDT antibacterial coatings on medical devices against MDR bacterial infections.

## 2 Results and Discussion

### 2.1 Characterization, Photothermal, and Photodynamic Properties of BDP‐6

**Figure 1:**
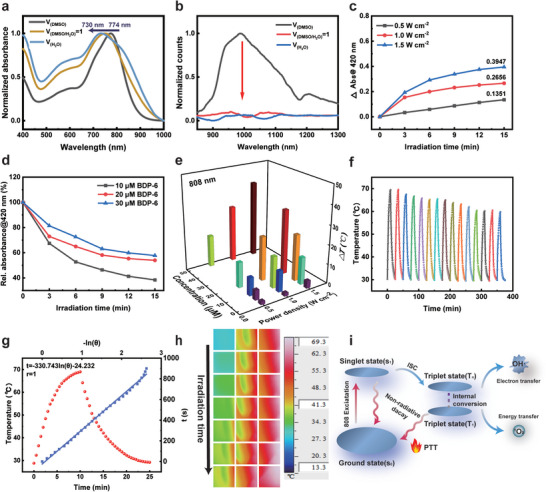
Synthesis and characterization of BDP‐6. a) The absorption spectra and b) fluorescence emission spectra of solutions of BDP‐6 in DMSO, *V*(DMSO/H_2_O) = 1 and H_2_O, respectively. Measurement of photosensitizing capabilities of c) BDP‐6 (10 µm) at different power densities (0.5, 1.0, and 1.5 W cm^−2^) and d) different concentrations BDP‐6 (10, 20, and 30 µm) at 1.5 W cm^−2^ in water via recording the absorbance changes of DPBF at 420 nm upon light irradiation, respectively. e) Evaluation of photothermal capabilities of BDP‐6 in different concentration solutions upon light irradiation at different power densities (0.5, 1.0, and 1.5 W cm^−2^), respectively. f) Temperature changes recorded for aqueous solutions of BDP‐6 (50 µm) during 15 cycles of 808 nm laser irradiation (1.5 W cm^−2^; 10 min per cycle). g) Temperature‐change curves of BDP‐6 with or without laser irradiation (808 nm, 1.5 W cm^−2^; 10 min) and linear fitting of −ln(*θ*) and time of BDP‐6. h) IR thermal images recorded for aqueous solutions of BDP‐6 (50 µm) upon 808 nm laser irradiation at 1.5 W cm^−2^ for 30 s intervals during 10 min. i) Schematic diagram of the photophysical process leading to reactive oxygen species (ROS) production and photothermal effect of BDP‐6.

The compound BDP‐6 was synthesized (Scheme S1, Supporting Information) and structurally characterized by ^1^H NMR and mass spectrometry (Figures S1–S10, Supporting Information). The absorption spectra of BDP‐6 in dimethyl sulfoxide (DMSO)/water with different water fractions were recorded to determine the H‐aggregation of BDP‐6 (**Figure** **1**a). In DMSO, BDP‐6 displayed a maximal NIR absorption peak (*λ*_max_) at 774 nm. After increasing the water fraction in the DMSO/water mixture to 50%, the aggregation of BDP‐6 by *π*–*π* and hydrophobic interactions leaded to a blueshift in the NIR absorption peak from 774 to 750 nm. A new blueshifted absorption band of BDP‐6 at 730 nm emerged in aqueous solution, manifesting the H‐aggregation of BDP‐6. The reasons for these phenomena were ascribed to the molecular structure characteristics of BDP‐6 and the solvent effect. On the one hand, the *n* → π* transition occurred between the *π*‐electrons on the formyl group (—CH=O) and the *n*‐orbital electrons of the oxygen atom. Meanwhile, the electrons in the *n*‐orbital of the oxygen atom could be solvated. After solvation, the oxygen atom increased the electron‐absorbing effect, reduced the energy of the n‐orbital of the ground state molecule, and increased the energy of *n* → π* transition. Thus, the absorption spectrum of BDP‐6 was blueshifted with the increase of solvent polarity. On the other hand, the polarity of solvent had a strong inducing effect on the conjugated structure of BDP‐6. As the polarity of the solvent increasing, the greater stabilizing effect on the excited state of BDP‐6 resulted in a shift of the fluorescence spectrum toward the long‐wave direction. The BDP‐6 presented a characteristic NIR‐II emission band ranging from 850 to 1200 nm in DMSO under 808 nm laser excitation, with a peak at 980 nm (Figure 1b). When the water content in the DMSO/water mixture was increased, aggregation‐caused quenching resulted in dramatic fluorescence quenching of BDP‐6, indicating that H‐aggregation significantly inhibited the singlet radiative decay pathway.

The photophysical and photochemical properties of BDP‐6 were elucidated by the density functional theory, which included the highest occupied molecular orbital (HOMO) and lowest unoccupied molecular orbital (LUMO) distributions and the ∆*E*_S1T1_ values of BDP‐6. As shown in Table S1 in the Supporting Information, BDP‐6 had a well‐separated HOMO and LUMO distributions, which was essential for obtaining a small ∆*E*_S1T1_. The small Δ*E*_S1T1_ value between singlet and triplet states of BDP‐6 favored intersystem crossing (ISC), which was a prerequisite for ROS generation. Then, electron paramagnetic resonance (EPR) spectroscopy was employed to distinguish ROS generation by sensitization of BDP‐6 upon 808 nm light irradiation (Figure S11, Supporting Information). 2,2,6,6‐tetramethylpiperide (TEMP) was applied as a spin trapper to identify singlet oxygen (^1^O_2_) and 5,5‐dimethyl‐1‐pyrroline‐*N*‐oxide (DMPO) was used as the spin‐trapping agents for superoxide radicals ($\left(\cdot O_{2}^{-}\right)$) or hydroxyl radicals (·OH). A significant set of EPR signals was observed when irradiating solution of BDP‐6 and TEMP, which was attributed to 2,2,6,6‐tetramethylpiperidin‐1‐oxyl formation, confirming singlet oxygen (^1^O_2_) generation. Meanwhile, a weak signal of the characteristic paramagnetic adduct was observed and matched with the ·OH signal in DMPO solution, indicating the production of ·OH. Based on the above results, we focused on the properties of singlet oxygen (^1^O_2_) for BDP‐6.

Thus, the ^1^O_2_ generation of BDP‐6 was measured by using 1,3‐diphenylisobenzofuran (DPBF) as a singlet oxygen probe under 808 nm laser irradiation. As shown in Figure 1c, the absorption intensity of DPBF was proportional to the laser power density under the same concentration of BDP‐6 (10 µm) (Figure S12a–c, Supporting Information). However, the decrease of absorption intensity of DPBF slowed down with the increase of concentrations of BDP‐6 aqueous solutions under laser irradiation with the same power density. It was assumed that increasing concentration of BDP‐6 aqueous solution could lead to further aggregation and ^1^O_2_ quenching, which might be beneficial to promote nonradiative transition and enhance photothermal properties (Figure 1d and Figure S12d–f, Supporting Information). To quantify the ^1^O_2_ generation efficiency of BDP‐6, DPBF was used as the ^1^O_2_ probe, and the indocyanine green (ICG) as the standard photosensitizer (PS) (Φ_ICG_ = 0.2%). The experiment used NIR laser at 808 nm. The absorbance intensity of DPBF gradually decreased with time under NIR light at 1.5 W cm^−2^, implying that DPBF was decomposed by the production of ^1^O_2_ (Figure S13, Supporting Information). Meanwhile, the decomposition rate constants for DPBF by BDP‐6 was 0.1187 min^−1^, which was larger than that of ICG (*K*_ICG_ = 0.04952 min^−1^). Thus, the ^1^O_2_ quantum yield of BDP‐6 can be calculated to be 0.38%, which was slightly higher than the value of ICG. These results indicate that BDP‐6 is an excellent photosensitizer for photodynamic therapy.

In addition, we evaluated the photothermal performance of BDP‐6 aqueous solution (Figure 1e). After 10 min irradiation by 808 nm laser, the distinct photothermal effect of BDP‐6 was clearly visualized through the quantitative temperature change. As the concentration of BDP‐6 solution increased, the temperature increased more obviously under the laser irradiation with the same power density (Figure S14, Supporting Information). Moreover, the temperature of BDP‐6 at high power density was significantly higher than that at lower power density under the same concentration of laser irradiation (808 nm, 10 min) (Figure S15, Supporting Information). Thus, the photothermal effect was positively related to the BDP‐6 concentration as well as laser exposure power density and irradiation time, indicating that BDP‐6 had the efficient photothermal effect.

Photothermal stability is an essential requirement for phototherapy in vivo.^[^ ^50^ ^]^ Gratifyingly, the temperature elevation of the BDP‐6 solution was still higher than 60 °C during 15 cycles of 808 nm laser irradiation (1.5 W cm^−2^, every cycle: 10 min), which was efficient to inactivate bacteria (Figure 1f). This result demonstrated good photothermal stability of BDP‐6. Then, the PCE of BDP‐6 was tested by heating and cooling curves of BDP‐6 solution (Figure 1g). Unsurprisingly, BDP‐6 showed a high photothermal conversion ability with the PCE value up to 55.59%. The strong NIR photothermal effect of BDP‐6 could be clearly seen by infrared thermal imaging (Figure 1h). On the basis of the above findings, the ^1^O_2_ generation properties and photothermal performance of BDP‐6 could be explained by the schematic diagram of photophysical process (Figure 1i). The excellent photothermal and photodynamic properties made BDP‐6 an effective candidate of PTT/PDT antibacterial component against MDR bacterial infection.

### 2.2 Physical and Chemical Properties of BDP‐6‐Based Coating‐Functionalized PU (PU‐GHB)

**Figure 2:**
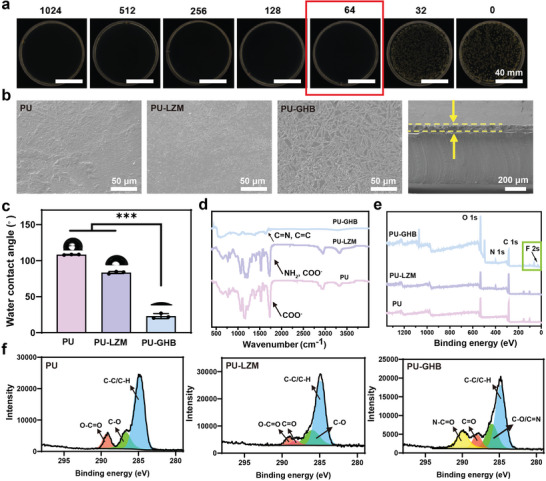
Physical and chemical properties. a) Minimum inhibitory concentration (MIC) of BDP‐6 against MRSA. b) SEM images and c) water contact angles of PU, PU‐LZM, and PU‐GHB. d) ATR‐FTIR spectra and e) XPS wide‐scan spectra of PU, PU‐LZM, and PU‐GHB. f) C 1s core‐level spectra of PU, PU‐LZM, and PU‐GHB. Data are presented as means ± SD of *n* = 3. The *p* values are calculated using two‐tailed unpaired *t*‐test, *p < 0.05, **p < 0.01, and ***p < 0.001.

The above results proved the BDP‐6 could produce ROS and hyperthermia under NIR irradiation, which might be facilitated for bactericidal application. Therefore, the antibacterial properties of BDP‐6 were evaluated first. To explore the dosage of BDP‐6 in surface coatings, the bactericidal activity of BDP‐6 was measured. The BDP‐6 solutions with different concentrations were incubated with a typical species of MDR bacteria, MRSA, after 10 min of NIR irradiation at the power density of 1.2 W cm^−2^. The amounts of the bacteria were significantly reduced when the concentrations of BDP‐6 were over 64 µg mL^−1^ (Figure S16, Supporting Information). Thus, the minimum inhibitory concentration (MIC) of the BDP‐6 was calculated as 64 µg mL^−1^ (**Figure** **2**a).

The free BDP‐6 molecules tend to clump together in the aqueous solution, which might reduce the photodynamic efficiency. However, the accumulation of BDP‐6 could be avoided in the coating due to the hydrogen bonding and binding forces, which could improve the photodynamic effect. Meanwhile, natural polymers (OHA and gelatin) were used to combine the BDP‐6 to modify the surface of PU. For determining the ratio of each component of the coatings, the amounts of reactive functional groups of each component were quantified. The content of amino group in gelatin was quantified as 32.16 µg/100 mg by the method of ninhydrin (Figure S17, Supporting Information). The oxidation degree of OHA was measured as 9.85% (Figure S18, Supporting Information). First, the surface of the PU was modified with lysozyme (LZM) to provide reaction sites,^[^ ^51^ ^]^ which was denoted as PU‐LZM. Then, the pre‐coated solution was prepared with 100 mg gelatin, 50 mg OHA and 2 mg BDP‐6 according to the ratio of amino and aldehyde groups, in which the ratio of NH_2_ (gelatin): CHO (OHA): CHO (BDP‐6) was 32.16:25:9. At last, the samples were dried slowly at 45 °C to form coating‐functionalized PU (PU‐GHB).

To characterize the physical and chemical properties of PU‐GHB, the samples were analyzed by scanning electron microscopy (SEM), water contact angles (WCA), attenuated total refraction Fourier transform infrared (ATR‐FTIR) spectroscopy, and the X‐ray photoelectron spectroscopy (XPS). The surface morphologies and the thickness of the coating were observed by SEM. A black coating could be observed after surface functionalization (Figure S19, Supporting Information). As shown in Figure 2b, the morphologies of the PU‐GHB could be observed, which appeared bamboo leaf‐like structures. And the thickness of the coating was ≈60 µm, which was uniform and flat. The OHA with low oxidation degrees retained the large amount of hydroxyl groups, which kept the excellent hydrophilicity of PU‐GHB. Therefore, the water contact angle of the coating decreased significantly (Figure 2c), which could be beneficial for antifouling performance of the coating.

The surface chemical compositions of PU‐GHB were characterized by ATR‐FTIR and XPS (Figure 2d–f). In the ATR‐FTIR spectra, the peaks at 1730 and 1628 cm^−1^ were assigned to the stretching vibration of C=O and C=N/C=C bonds, respectively. The C=N bonds indicated the coatings were fabricated by Schiff base reaction. Meanwhile, the XPS spectra indicated the presence of F element, which was attributed to BDP‐6. This result proved the BDP‐6 was integrated into the PU‐GHB coating successfully. The N—C=O bond was attributed to OHA and gelatin. Also, the C 1s signals at the binding energy of 286 eV of PU‐GHB were attributed to the C=N bond in the Schiff base structure. The above results proved that PU‐GHB was prepared successfully.

### 2.3 In Vitro Antibacterial Performances and Antibacterial Mechanism

**Figure 3:**
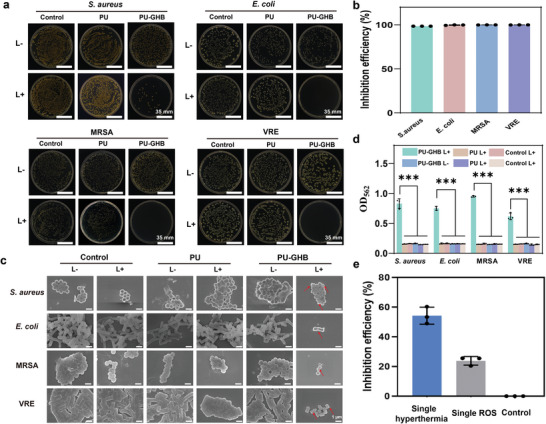
Antibacterial analysis. a) Bacterial colony counting and b) the antibacterial efficiencies against *S. aureus*, *E. coli*, MRSA, and VRE. c) SEM images of the morphologies of bacteria that were treated PU, PU‐LZM, and PU‐GHB with and without 808 nm NIR light irradiation. d) Protein leakage of *S. aureus*, *E. coli*, MRSA, and VRE. e) The antibacterial effect of single hyperthermia or ROS. Data are presented as means ± SD of *n* = 3. The *p* values are calculated using two‐tailed unpaired *t*‐test, *p < 0.05, **p < 0.01, and ***p < 0.001.

In view of that BDP‐6 could produce ROS and hyperthermia with 808 nm NIR irradiation as mentioned above, the excellent antibacterial performance of BDP‐6 alone had been verified in vitro (Figure 2a). To confirm the antibacterial performance of PU‐GHB, four different species of bacteria (S*. aureus*, *E. coli*, MRSA, and vancomycin‐resistant *Enterococcus* (VRE)) were chosen (**Figure** **3**a). After 808 nm NIR irradiation at the power density of 1.2 W cm^−2^ for 10 min, the surface temperature was increased by 39.7 °C (Figure S20, Supporting Information) and could produce ROS (Figure S21, Supporting Information), which could be utilized for combined PTT/PDT bactericidal effects. In the meantime, the photodynamic effect of the BDP‐6 in PU‐GHB was higher than the equimolar concentration of the free BDP‐6 molecules (Figure S22, Supporting Information). The mesophilic bacteria viability can be altered by increasing the temperature above 45 °C.^[^ ^52^ ^]^ Thus, the antibacterial effects of PU‐GHB were prominent after NIR irradiation (L+), which inactivated over 99% of bacteria, including MDR bacteria (Figure 3b). On the contrary, the antibacterial activity of PU‐GHB without NIR irradiation was similar to the PU and control groups. The results implied that the antibacterial activity of PU‐GHB was triggered by NIR stimulation. Meanwhile, live/dead fluorescent staining was utilized to observe the viabilities of planktonic bacteria in different groups. The live bacteria showed green fluorescence (Figure S23, Supporting Information). The results were consistent with the data of bacterial culture (Figure 3a). PU‐GHB without NIR irradiation and PU showed poor antibacterial property. In contrast, few of live bacteria could be observed in the PU‐GHB (L+) group, indicating the high and broad‐spectrum antibacterial effects.

To explore the antibacterial mechanism of PU‐GHB, the bacterial morphologies were observed by SEM first (Figure 3c). The four species of bacteria that were treated by PU‐GHB under NIR irradiation showed membrane distortion, wrinkled surface, and content leakage, which were contributed by the actions of hyperthermia and ROS. In other groups, all bacteria showed smooth and integrated surface without any damage. These results demonstrated that the PU‐GHB under 808 nm NIR irradiation could reduce the bacterial activity by disturbing the membrane structure of bacteria in a short time. Then, the evaluation of protein leakage was performed to confirm the damaging effect of PU‐GHB on cell membrane (Figure 3d). After NIR irradiation, the amounts of protein leakage in the PU‐GHB group increased obviously in all species of bacteria. However, there was no significant difference in the amount of protein leakage in other groups. The results of morphology and protein leakage showed that PU‐GHB under irradiation of 808 nm NIR could lead to cell membrane disruption and leakage of intracellular contents on both susceptible and MDR bacteria, which were facilitated for efficient and broad‐spectrum antibacterial performances.

To verify the synergistic antibacterial effect between hyperthermia and ROS, the single hyperthermia or the single ROS antibacterial effect were tested against MRSA. As shown in Figure 3e, the antibacterial efficiency with single hyperthermia was 54% and that with single ROS was 24%. In contrast, the combined PTT/PDT antibacterial effect against MRSA was >99.99% (Figure 3b). These results showed the synergistic PTT/PDT antibacterial effect of PU‐GHB could achieve a better antibacterial performance than single modal antibacterial effect. The hyperthermia could reduce the bacterial activity by disturbing the cell membrane structure, and enhance the inner membrane permeability to ROS,^[^ ^53^ ^]^ which further aggravated the damage of cell membrane and protein leakage.

**Figure 4:**
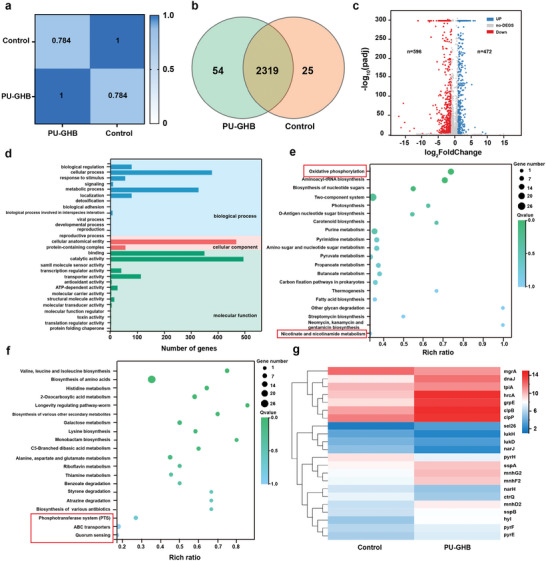
Gene transcription analysis. a) Pearson's correlations between PU‐GHB and control group. b) Venn diagram of the number of DEGs. c) Volcano plots of DEGs, |log2 fold change| > 1 and padj < 0.001 were used as the cutoff for significant DEGs. d) Gene ontology (GO) annotation analysis of DEGs. e) Upregulated and f) downregulated DEGs enriched in the Kyoto Encyclopedia of Genes and Genomes (KEGG pathway (entries with larger bubbles contain more DEGs). g) Heatmap of genes associated with interspecies interaction.

To further understanding the antibacterial mechanism of PU‐GHB, we performed gene transcription analysis of PU‐GHB‐treated MRSA with 808 nm NIR irradiation for 10 min at the power density of 1.2 W cm^−2^. The MRSA without any treatment was taken as a control group. The differentially expressed genes (DEGs) in different groups were assessed using Pearson's correlation coefficients. According to the encode criteria, the square Person coefficient (*R* ^2^) of correlation for the replicatable biological samples should be at least 0.92. However, the *R* ^2^ between the PU‐GHB and control groups was 0.784, which indicated the expressed genes of MRSA was regulated by PU‐GHB under NIR irradiation (**Figure** **4**a). In the gene transcription analysis of MRSA, 2398 genes were shared by two groups, while 54 genes were expressed exclusively in the PU‐GHB group (Figure 4b). The volcano plots showed that 1068 genes expressed differentially between two groups, with 472 upregulated genes and 596 downregulated genes (Figure 4c). To further clarify the effects of PU‐GHB on MRSA's biological functions, we performed gene ontology (GO) analysis. The DEGs in GO terms were belonged to biological process, cellular component and molecular function (Figure 4d). The enrichment analysis of Kyoto Encyclopedia of Genes and Genomes (KEGG) pathways (Figure 4e,f) showed that the DEGs associated with antiheat‐shock, antioxidative‐stress function and energy metabolism were stimulated by PU‐GHB.

Many upregulated DEGs related to heat shock stress, such as *clpB*, *dnaJ*, and *grpE*, which are associated with the production of HSPs (Figure 4g). HSPs, a series of chaperones, could help the cell to resist thermal shock and maintain cell activity under elevated ambient temperatures. Among them, HSP70 and HSP90 are major inducers in PTT antimicrobial strategies.^[^ ^9^ ^]^ The upregulated heat‐shock‐related genes suggested that PU‐GHB induced MRSA to produce HSP inducers and react to the changes in the environmental temperature as a response to PTT. The expression of MRSA virulence determinants was influenced by a variety of regulatory influences. The gene encoding important regulators including *hyl*, *sel26*, and *mgrA* were downregulated in MRSA after PU‐GHB treatment. The downregulated virulent genes might benefit the host to eliminate MRSA infection. These results further revealed the antibacterial mechanisms of PU‐GHB.

### 2.4 In Vivo Photothermal Anti‐Infection Therapeutic Performances in a Hernia Animal Model

The PU‐GHB showed outstanding in vitro antibacterial performances, which demonstrated PU‐GHB could be used for in vivo anti‐infection study. In clinic, a large number of medical device‐related infection were caused by poor infection prevention and misuse of antibiotics after operation.^[^ ^54^, ^55^ ^]^ Among these, the infection caused by MDR bacteria was more difficult to treat. Thus, in order to evaluate anti‐infection performances of PU‐GHB in clinical setting, MRSA‐infected hernia animal model was built in Sprague‐Dawley (SD) mice. All of the animal experiments were performed in compliance with the guidelines issued by the Ethical Committee of the Chinese Academy of Sciences. For the comprehensive study of anti‐infection properties of PU‐GHB, all SD mice were divided into six groups in accordance with different samples, with/without bacteria (B+, B−) and with/without NIR irradiation (L+, L−) (Figure S24, Supporting Information). The L+ groups were irradiated by 808 nm NIR at the power density of 1.0 W cm^−2^ for 15 min. The temperature of the PU‐GHB group increased by 18.3 °C (Figure S25, Supporting Information), indicating PU‐GHB still had photothermal effects inside the abdominal wall.

**Figure 5:**
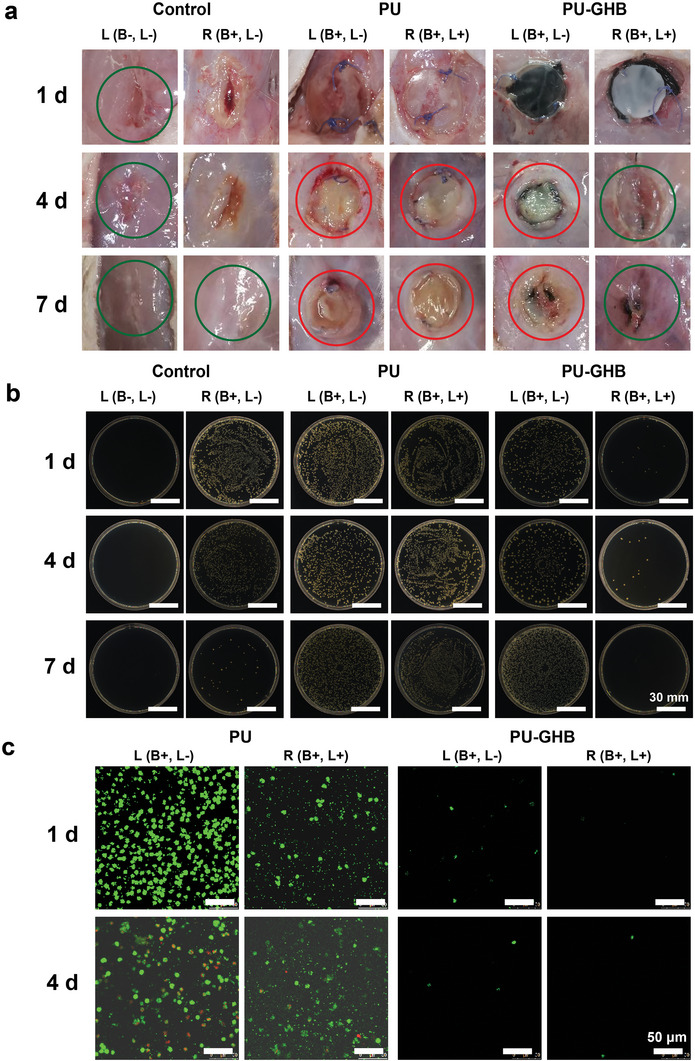
General views and anti‐infection properties in vivo. a) Views of the infected areas of abdominal wall hernia model. b) Images of bacterial colonies from the tissues in the surgical sites. c) CLSM images of the sample surfaces at 1 and 4 d.

To evaluate the in vivo antibacterial properties of PU‐GHB, the tissues around the samples were observed from both macroscopic and microscopic perspectives. First, the mice in each group were sacrificed to observe from macroscopic perspectives at 1, 4, and 7 d. From the general view, the mice in the control (B−) and PU‐GHB (L+) groups recovered well, indicating the mice in the PU‐GHB (L+) group were not affected by bacterial infection (**Figure** **5**a). In contrast, severe purulency could be observed in the PU (L+, L−) and PU‐GHB (L−) groups. Meanwhile, the high efficiency in minimizing adhesion‐related complications and integration by surrounding tissues is a key characteristic required from soft tissue repair materials.^[^ ^56^ ^]^ In the PU‐GHB (L+) group, the tissue was well integrated under the mesh. However, in the other groups, the mesh was encased in the surrounding tissue and with adhesion. The above‐mentioned results indicated that PU‐GHB could play a good role in antiadhesion property and tissue integration, which was due to its good hydrophilic properties, biocompatibility, and antibacterial properties.

The tissue around the samples was harvested for bacterial culture (Figure 5b). After 1 d, the average number of the bacterial colonies in PU‐GHB (L+) was significantly lower than those in other groups, which proved PU‐GHB (L+) had excellent antibacterial effect in vivo. In addition, to further verify the anti‐infection effect of PU‐GHB, the samples after live/dead staining were observed by confocal laser scanning microscope (CLSM) (Figure 5c). A large number of bacteria and cells could be seen in PU group, indicating severe infection and inflammation occurred. In contrast, there were few bacteria that adhered on the surface of PU‐GHB, which showed the high ability to reduce the possibility of potential infection. The above‐mentioned results indicated PU might provide the space for bacterial colonization to aggravate infection, while PU‐GHB could eliminate bacteria and prevent infection.

**Figure 6:**
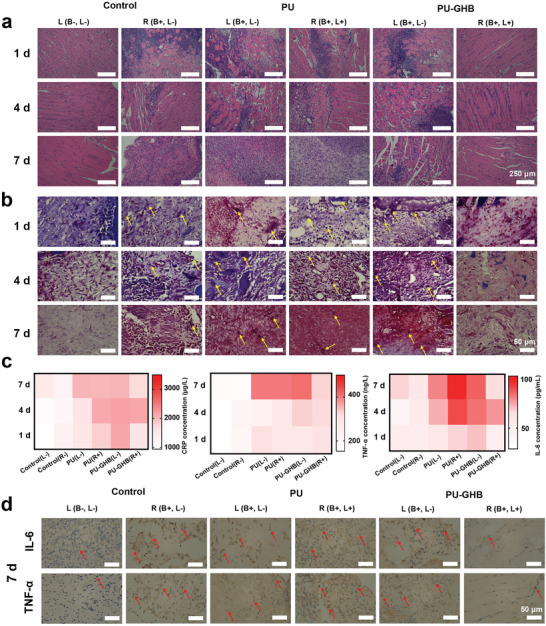
Histological analysis. a) H&E and b) Gram's staining images of the tissues around the samples at 1, 4, and 7 d (yellow arrows indicating bacteria). c) The levels of CRP, TNF‐*α* and IL‐6 in the tissues around the samples at 1, 4, and 7 d. d) The immunohistochemical staining images of IL‐6 and TNF‐*α* at 7 d after operation (red arrows indicating positive area).

The macroscopic observation preliminarily confirmed that PU‐GHB had good anti‐infection performance in vivo. In order to further verify the anti‐infection property of PU‐GHB, we histologically observed the tissues around the samples from microscopic perspective. The infected tissues around the samples were analyzed by hematoxylin and eosin staining (H&E, **Figure** **6**a) and Gram's staining (Figure 6b) at 1, 4, and 7 d after operation. By the histological images, severe inflammatory cell infiltration was observed in the muscle tissues of the control (B+, L−), PU (B+, L+ and B+, L−) and PU‐GHB (B+, L−) groups. In the PU‐GHB (B+, L+) group, few inflammatory cells were observed. What is more, the tissue irritation of PU‐GHB was also verified by the PU‐GHB (B−, L+) group, in which PU‐GHB samples were implanted without bacteria and NIR irradiation. In the tissues of the PU‐GHB (B−, L+) group, few inflammatory cells were observed (Figure S26, Supporting Information), which demonstrated the low tissue irritation. Meanwhile, Gram's staining was performed to verify whether the inflammatory response was caused by bacteria infection. No bacteria were found in the control (B−, L−) and PU‐GHB (B+, L+) groups. However, a large number of bacteria were observed in other groups (indicated by yellow arrows) in the area of the infected tissues at 1, 4, and 7 d after operation, indicating severe bacterial infection. These results suggested that PU‐GHB could eliminate bacteria in the tissue and inhibit infection under NIR irradiation efficiently, while it also possessed good histocompatibility.

Bacterial infection would attack the wound and aggravate inflammation.^[^ ^57^ ^]^ To determine the inflammatory response quantitatively, the levels of three inflammatory indicators, C‐reactive protein (CRP), interleukin 6 (IL‐6), and tumor necrosis factor *α* (TNF‐*α*), were tested (Figure 6c). IL‐6 can reflect the intensity of infection directly.^[^ ^58^ ^]^ TNF‐*α* is a proinflammatory cytokine in the early stages of infection, which shows high sensitivity to bacterial infection.^[^ ^59^ ^]^ The CRP values of the PU‐GHB (B+, L+) increased first and then backed to normal, indicating that the infection was inhibited in the PU‐GHB (B+, L+) group. In contrast, the CRP values of the PU (B+, L+), PU (B+, L−), and PU‐GHB (B+, L−) groups kept increasing significantly after operation. The IL‐6 and TNF‐*α* values of the PU (B+, L+), PU (B+, L−), and PU‐GHB (B+, L−) groups still maintained a high level at 7 d, but those of the PU‐GHB (B+, L+) were in a lower level. These results meant that PU‐GHB had notable anti‐infection performances and could decrease inflammatory reaction obviously.

For further determination of the distribution of the inflammatory reaction in the tissues, two kind of typical inflammatory factors, IL‐6 and TNF‐*α*, were visualized by immunohistochemical staining (Figure 6d and Figure S27, Supporting Information). From the immunohistochemical images, the inflammatory reaction areas appeared bright yellow. The positive areas of IL‐6 and TNF‐*α* of the PU‐GHB (B+, L+) group were lower than other groups. The results were consistent with the quantitative results of the inflammation factors in the tissues, which continually indicated the excellent anti‐infection performances of PU‐GHB under NIR irradiation.

### 2.5 Biocompatibility and Photoacoustic Imaging

**Figure 7:**
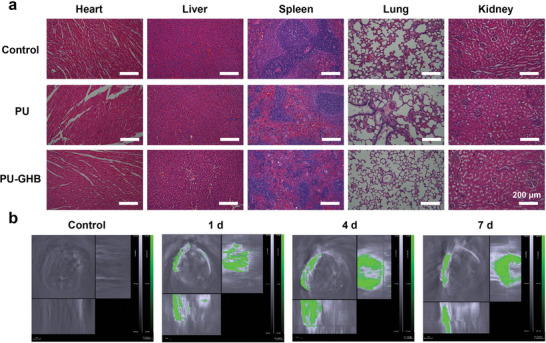
Biocompatibility analysis and photoacoustic imaging. a) The H&E staining images of the hearts, livers, spleens, lungs, and kidneys of different groups at 7 d after operation. b) The photoacoustic (PA) images of PU‐GHB at 1, 4, and 7 d.

Good biocompatibility is an important characteristic of implanted biomedical devices. The good in vitro biocompatibility of PU‐GHB had been proven by 3‐(4,5‐dimethyl‐2‐thiazolyl)‐2,5‐diphenyl‐2‐H‐tetrazolium bromide (MTT) method with L929 fibroblast cells, and it could affect the viability of mammalian cells with NIR irradiation (Figure S28, Supporting Information). To evaluate the in vivo biocompatibility, the main organs (heart, liver, spleen, lung, and kidney) of the experimental animals were harvested and analyzed by H&E staining. The H&E staining images showed that the organs exhibited normal state in all groups (**Figure** **7**a). The results of histological analysis of main organs and local tissues (Figure S26, Supporting Information) revealed the good in vivo biocompatibility of the PU‐GHB despite the in vitro influence on cell viability. The above results proved that PU‐GHB had high biocompatibility both in vitro and in vivo, which could be used for antibacterial functionalization of implanted medical devices.

Besides, the BDP‐6 not only could be used as an antibacterial agent but also might be used as a theragnostic agent for PA imaging on account of its absorbance peak at 725 nm, which could visualize PU‐GHB in vivo. The PA image diagnose had a locating function to determine whether the PU‐GHB had displacement and shedding phenomena. As displayed in Figure 7b, the strong PA signal at the abdomen region was observed at 1, 4, and 7 d after operation, which showed the position and shape of the PU‐GHB samples inside living bodies. The results showed no displacement and shedding phenomena occurred in the PU‐GHB group. From this perspective, PU‐GHB had satisfactory PA imaging performance in vivo while providing excellent anti‐infection effect. On the basis of the above findings, this work could provide much possibilities for constructing multifunctional imaging and therapeutic platforms on implanted medical devices.

## 3 Conclusions

In this work, we successfully synthesized a new heavy atom free photosensitizer compound, BDP‐6, without complex covalent decoration. BDP‐6 exhibited long‐wavelength absorption, high PCE, and high photostability, which could be used as a dual‐mode PTT/PDT therapeutic agent. By combination with natural polymers, the BDP‐6 molecules were successfully integrated into the surface of PU to construct antibacterial functionalization coating (PU‐GHB). PU‐GHB could efficiently convert NIR light into hyperthermia and ROS and then achieve rapid and efficient antibacterial activities against both susceptible bacteria and MDR bacteria. Mechanism studies revealed that hyperthermia promoted the production of ROS, and both hyperthermia and ROS could disturb the cell membrane and metabolism of the bacteria synergistically. Moreover, in vitro and in vivo toxicity evaluations proved the good biocompatibility of the PU‐GHB. On the basis of these performances, PU‐GHB could effectively eradicate MDR bacteria in an infected abdominal wall hernia model. Meanwhile, the in vivo PA imaging property of PU‐GHB was verified, which provided a tool for real‐time visualization of implanted medical device in living bodies. This work provides a productive strategy to eradicate MDR bacterial infection through the BDP‐6‐based PTT/PDT synergetic therapy, which builds a solid foundation for the development of multifunctional antibacterial medical devices.

## Conflict of Interest

The authors declare no conflict of interest.

## Supporting information

Supporting Information

## Data Availability

The data that support the findings of this study are available from the corresponding author upon reasonable request.
