# Supplementary material for: BODIPY‐Functionalized Natural Polymer Coatings for Multimodal Therapy of Drug‐Resistant Bacterial Infection
Source: Adv Sci (Weinh). 2023 Mar 19;10(14):2300328. doi: 10.1002/advs.202300328 (PMC10190636; doi:10.1002/advs.202300328)
Supplement: Supplementary file 1 — Supporting Information [file ADVS-10-2300328-s001.pdf]

ADVANCED SCIENCE
Open Access

Supporting Information

for *Adv. Sci.*, DOI 10.1002/advs.202300328

# BODIPY-Functionalized Natural Polymer Coatings for Multimodal Therapy of Drug-Resistant Bacterial Infection

*Lujiao Zhang, Chenyan Hu, Meizhou Sun, Xiaokang Ding, Hong-Bo Cheng*, Shun Duan* and Fu-Jian Xu**

# SUPPORTING INFORMATION

# BODIPY-Functionalized Natural Polymer Coatings for Multimodal Therapy of Drug-Resistant Bacterial Infection

Lujiao Zhang[a], Chunyang Hu[a], Meizhou Sun, Xiaokang Ding, Hong-Bo Cheng[*], Shun Duan[*], Fu-Jian Xu[*]

State Key Laboratory of Chemical Resource Engineering, State Key Laboratory of Organic-Inorganic Composites, Key Lab of Biomedical Materials of Natural Macromolecules (Beijing University of Chemical Technology), Ministry of Education, Beijing Laboratory of Biomedical Materials, Beijing 100029, China

E-mail addresses: chenghb@mail.buct.edu.cn (H.B.C.); duanshun@mail.buct.edu.cn (S.D.); xufj@mail.buct.edu.cn (F.J.X.)

## 1. Experimental Section

### 1.1 Materials

For the synthesis of the BDP-6, all reagents were purchased from Beijing Innochem Science & Technology or J&K Scientific unless otherwise stated. Both 2,2,6,6-tetramethylpiperide (TEMP) and 5,5-dimethyl-1-pyrroline-N-oxide (DMPO) were purchased from J&K Scientific. Indocyanine green (ICG) was purchased from Beijing Innochem Science & Technology. 1,3-diphenylisobenzofuran (DPBF) was purchased from APE Bio. For the surface modification process, lysozyme and gelatin were purchased from Sigma-Aldrich (USA). Sodium bicarbonate ($NaHCO_3$), anhydrous sodium carbonate ($Na_2CO_3$) and sodium hydroxide (NaOH) were purchased from Beijing Chemical Works (China). 2-[4-(2-hydroxyethyl)-1-piperazinyl] ethanesulfonic acid (HEPES) was purchased from TCI Chemical Industry (China). Hyaluronic acid (HA), sodium periodate ($NaIO_4$), ethylene glycol (EG), hydroxylamine hydrochloride and tris(2-chloroethyl) phosphate (TCEP) were purchased from Energy Chemical (China).

### 1.2 Methods

$^1$H-NMR spectra of all compounds were performed with Bruker Avance Ⅲ 400

MHz spectrometer. HRMS (ESI) data were carried out using Waters ultra-performance liquid chromatography quadrupole time of flight tandem mass spectrometry (Xevo G2 Qtof, USA). UV-vis spectra were recorded by SHIMADZU U3600 spectrometer (SHIMADZU, Japan). The steady-state fluorescence spectra were measured on a FLS980 Series of Fluorescence Spectrometers (edinburgh instrument, UK). Electron paramagnetic resonance (EPR) spectra were collected with a Bruker EPR spectrometer (EMX-500 10/12, Germany). The diameter of the nanoparticles was determined by dynamic light scattering (DLS) on a Zetasizer Nano ZS90 (Malvem, UK). Photoacoustic imaging was carried out by the InVision 256-TF MSOT system (iTheraMedical, Germany). A laser at $\lambda$ = 808 nm was employed as the light source for *in vitro* and *in vivo* experiments. The output power of the laser was controlled by a fiber coupled laser system (FC-W-808-30W, Changchun New Industries Optoelectronics Technology) and measured by a power meter (CEL-NP2000, Beijing Zhongjiao Jinyuan Technology Co., Ltd.).

### 1.3 Synthesis Section

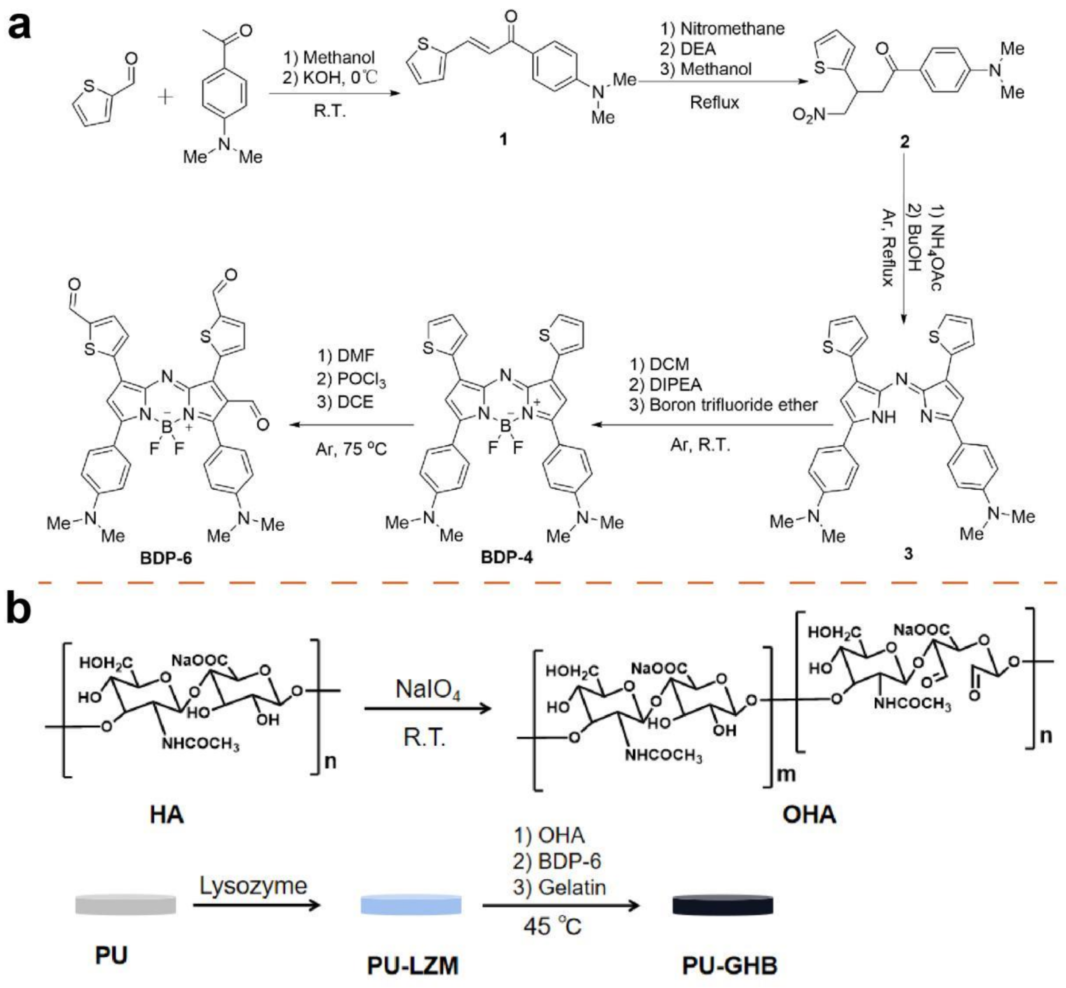


**Scheme S1.** a) Synthetic routes employed for the preparation of BDP-6; b) schematic illustration for the preparation of PU-GHB implant.

## Synthesis of Compound 1

2-Thiophenecarboxaldehyde (6.72 g, 5.43 mL, 59.92 mmol, 1.2 eq.) and 1-[4-(dimethylamino) phenyl]-ethanone (8.15 g, 49.93 mmol, 1 eq.) were dissolved in 250 mL methanol with 500 mL round bottom flask. After the mixture was cooled to 0 ℃, the aqueous potassium hydroxide solution (150 mL, 2.5 M) was added dropwise to the stirred reaction solution. Then, the reaction mixture was allowed to warm up to room temperature and stirred overnight. After cooling to 0 °C, the mixture was filtered, and the residue was washed with petroleum ether. The crude product was recrystallized from ethanol to obtain **1** as a yellow solid (9.79 g, 76.19%). $^1$H NMR (400 MHz, Chloroform-*d*): δ (ppm) 8.00-7.97 (m, 2H), 7.91 (d, J = 15.3 Hz, 1H), 7.42-7.35 (m, 2H), 7.32 (d, J = 3.6 Hz, 1H), 7.07 (dd, J = 5.1, 3.6 Hz, 1H), 6.70 (d, J =

9.1 Hz, 2H), 3.08 (s, 6H). HRMS (ESI) m/z $[M+H]^+$ was calculated for $C_{15}H_{15}NOS$: 258.0953, found: 258.0960.

**Synthesis of Compound 2**

Nitromethane (7.12 g, 6.26 mL, 116.57 mmol, 5 eq.), **1** (6.00 g, 23.31 mmol, 1 eq.) and diethylamine (8.53 g, 12.01 mL, 116.57 mmol, 5 eq.) were dissolved in 150 mL methanol with 250 mL round bottom flask, and heated in an oil bath until reflux overnight. After the solution was cooled to 0 °C, an aqueous hydrochloric acid (2.5 M) was added dropwise to the reaction solution until the pH was 2-3. Then methanol was removed under reduced pressure, and the crude product was dissolved with $CH_2Cl_2$. The organic phases were washed with brine and water, left to stand for separation, and the organic phases were dried over $Na_2SO_4$, evaporated in vacuo and chromatographed with silica gel. Elution with a 1:5 petroleum ether/ dichloromethane mixture afforded **2** as a deep yellow oil (3.13 g, 42.19%). $^1$H NMR (400 MHz, Chloroform-*d*): δ (ppm) 7.86 (d, J = 9.1 Hz, 2H), 7.20 (dd, J = 5.0, 1.2 Hz, 1H), 6.96-6.91 (m, 2H), 6.68 (d, J = 8.2 Hz, 2H), 4.87 (dd, J = 12.5, 5.8 Hz, 1H), 4.68 (dd, J = 12.5, 8.1 Hz, 1H), 4.54 (ddd, J = 13.8, 7.9, 6.0 Hz, 1H), 3.40 (dd, J = 12.3, 6.9 Hz, 2H), 3.07 (s, 6H). HRMS (ESI) m/z $[M+H]^+$ was calculated for $C_{16}H_{18}N_2O_3S$: 319.1116, found: 319.1123.

**Synthesis of Compound 3**

Ammonium acetate (16.95g, 219.86 mmol, 35 eq.) was added to a stirred solution of compound **2** (2 g, 6.28 mmol, 1 eq.) in 150 mL of n-BuOH in a two-necked round bottom flask under argon atmosphere, and the reaction solution was heated to reflux for 24 h. The reaction was cooled to room temperature and the solvent was concentrated by rotary evaporation. The mixture was filtered, and the residue was washed three times with EtOH to obtain **3** as a black solid (1.97 g, 57.26%). $^1$H NMR (400 MHz, DMSO-$d_6$): δ (ppm) 7.91-7.87 (m, 6H), 7.67 (d, J = 5.0 Hz, 2H), 7.40 (s, 2H), 7.21 (dd, J = 5.0, 3.7 Hz, 2H), 6.94 (d, J = 8.9 Hz, 4H), 3.09 (s, 12H). HRMS (ESI) m/z $[M+H]^+$ was calculated for $C_{32}H_{29}N_5S_2$: 548.1943, found: 548.1935.

## Synthesis of Compound BDP-4

**3** (1.00 g, 1.83 mmol, 1eq.) was dissolved in 120 mL anhydrous $CH_2Cl_2$ in a two-necked round bottom flask under the protection of argon. The reaction solution was stirred at 0 °C for 20 min, then diisopropylethylamine (2.36 g, 3.18 mL, 18.26 mmol, 10 eq.) was added drop dropwise. The reaction solution was continued to be stirred for 30 minutes, followed by the addition of boron trifluoride ether complex (7.77 g, 6.94 mL, 54.77 mmol, 30 eq.), and the reaction was stirred at room temperature for 24 h. The reaction solution was washed with brine and water for three times and separated. The organic layer was dried by anhydrous $Na_2SO_4$, and the solvent $CH_2Cl_2$ was removed by rotary evaporation. The crude product was purified by column chromatography (petroleum ether/ethyl acetate (4/1, v/v)), resulting in a dark brown solid BDP-4 that glows red in sunlight. (0.83 g, 76.15%). $^1$H NMR (400 MHz, Chloroform-*d*): δ (ppm) 8.10 (d, J = 9.0 Hz, 4H), 7.90 (d, J = 3.2 Hz, 2H), 7.48 (d, J = 4.6 Hz, 2H), 7.20-7.15 (m, 2H), 6.99 (s, 2H), 6.79 (d, J = 7.6 Hz, 4H), 3.09 (s, 12H). HRMS (ESI) m/z $[M+H]^+$ was calculated for $C_{32}H_{28}BF_2N_5S_2$: 595.1962, found: 595.1929.

## Synthesis of Compound BDP-6

Compound BDP-6 was prepared according to the literature procedure.[S1,S2] A mixture of DMF (3 mL) and $POCl_3$ (3 mL) was stirred in an ice bath for 5 min under argon. After being warmed to room temperature, it was stirred for an additional 30 min. The mixture and BDP-4 (100 mg, 0.17 mmol) were added into dichloroethane (20 mL), and stirred for an additional 24 h at 75 ℃. The mixture was then cooled to room temperature and slowly poured into saturated aqueous $NaHCO_3$ under ice-cold conditions. After being warmed to room temperature, the mixture was stirred for 6 h. The organic layers were combined, dried over anhydrous $MgSO_4$, and evaporated in vacuo. The crude product was further purified using column chromatography (silica gel, petroleum ether/ethyl acetate=1:3, v/v) to give BDP-6 as a dull brown solid (23.4 mg, 20.5%). $^1$H NMR (400 MHz, Chloroform-*d*): δ (ppm) 10.14 (s, 1H), 9.98 (s, 1H), 9.68 (s, 1H), 8.31 (d, J = 3.9 Hz, 1H), 8.21 (d, J = 7.7 Hz, 3H), 8.07 (s, 1H), 7.80 (d, J

= 3.9 Hz, 2H), 7.63 (d, J = 4.8 Hz, 1H), 7.32 (s, 1H), 6.72 (d, J = 8.5 Hz, 2H), 3.13 (d, J = 22.1 Hz, 12H). HRMS (ESI) m/z $[M+H]^+$ was calculated for $C_{35}H_{28}BF_2N_5O_3S_2$: 679.1809, found: 679.1812.

**Synthesis of Oxidized Hyaluronic Acid (OHA)**

HA (1 g) was dissolved in 100 mL of deionized water, and 0.106 g of $NaIO_4$ was added to react in the dark for 24 h. After 24 h, EG with equal molar ratio of $NaIO_4$ was added to terminate the reaction for 1 h. The raw product was dialyzed and freeze-dried to obtain OHA. The degree of oxidation was calculated by hydroxylamine hydrochloride titration.[S3] According to the volumes of consumed NaOH, the degree of the oxidation was calculated as 9.85%.

**Preparation of BDP-6-Loaded Coating-Functionalized PU (PU-GHB)**

The lysozyme (2 mg $mL^{-1}$) was dissolved in 3 mL of HEPES buffer (10 mM, pH=7.2), and mixed with 3 mL of TCEP solution (20 mM, pH=7.0). The PU was incubated with lysozyme solution for 1 h and obtained PU-LZM. Gelatin (100 mg) was dissolved into 1 mL of deionized water at 60 ℃. 50 mg of OHA was dissolved into 1 mL of deionized water and added into gelatin solution. The BDP-6 (10 mg) was dissolved by ultrasound into 100 μL of DMSO. Then, 20 μL of above-mentioned BDP-6 solution was added into 1 mL of $Na_2CO_3$-$NaHCO_3$ buffer solution (0.1 mol $L^{-1}$, pH=10). The BDP-6 solution was added into the gelatin solution to obtain the pre-coated solution. The PU-LZM was put into the pre-coated solution and slowly dried at 45 ℃ to prepare PU-GHB.

**1.4 UV-Vis Absorption and the Steady-State Fluorescence Spectra of BDP-6**

The UV-vis absorption spectra of BDP-6 in organic solvents with different polarity were recorded by SHIMADZU U3600 spectrometer (SHIMADZU, Japan). And the absorption spectra for BDP-6 were measured in different ratios of DMSO and $H_2O$. The NIR-Ⅱ fluorescence spectrum of BDP-6 in different polarity organic solvents and different volume fractions of DMSO/$H_2O$ solution were tested on a

FLS980 Series of Fluorescence Spectrometers.

### 1.5 Electron Paramagnetic Resonance (EPR) Spectra Experiments

In this study, electron paramagnetic resonance (EPR) was employed to monitor the ROS signals generated from BDP-6 under 808 nm irradiation (1.2 W). 2,2,6,6-tetramethylpiperide (TEMP) was used as the spin-trapping agents for singlet oxygen (1O2), and 5,5-dimethyl-1-pyrroline-N-oxide (DMPO) was used as the spin-trapping agents for superoxide radicals ($\cdot O_2^-$) or hydroxyl radicals (·OH).[S4] BDP-6 and TEMP or DMPO were prepared as test samples in aqueous solution under illumination. The EPR spectroscopy was measured on a Bruker EPR spectrometer after irradiation.

### 1.6 The Singlet Oxygen ($^1O_2$) Detection and $^1O_2$ Quantum Yield Calculation

1,3-diphenylisobenzofuran (DPBF) was used as a singlet oxygen probe to measure singlet oxygen generation of BDP-6 under 808 nm laser irradiation. In the experiment, DMSO was used as solvent to prepare high concentration DPBF solution (3 mM) and BDP-6 solution (3 mM), respectively. BDP-6 at 10 μM was mixed with 50 μM DPBF in 3 mL $H_2O$ under ultrasonication for 30 s. The mixture was then irradiated by lasers at different power densities (0.5-1.5 W $cm^{-2}$) for a period of 3 min, respectively. Next, different concentrations of BDP-6 (10 μM, 20 μM and 30 μM) were irradiated with 808 nm laser with a power density of 1.5W $cm^{-2}$ for 3 min at a time, respectively. The absorption intensity of DPBF at 420 nm in the presence of BDP-6 was recorded every three minutes under the 808 nm laser irradiation by UV-vis spectrophotometer.

For $^1O_2$ Quantum yield measurement, DPBF was used as the $^1O_2$ probe and indocyanine green (ICG) as the reference ($\Phi_{ICG}$ = 0.2%).[S5] The $^1O_2$ Quantum yield of BDP-6 aqueous solution was studied with indirect method.[S6] 50 μL DPBF/DMSO solution (3 mM) was added to 3 mL of the BDP-6 and ICG aqueous solution, respectively. The mixed solutions were irradiated with 808 nm laser light (1.5 W $cm^{-2}$). The maximal absorption of BDP-6/ICG and mixed solution were adjusted to about 0.2 OD and 1.0 OD, respectively. The absorption spectra of the mixed solution from 300

to 600 nm were recorded every 2 minutes to obtain the decay rate of the photosensitizing process. The $^1O_2$ Quantum yield ($\Phi_\triangle$) of BDP-6 was calculated according to the following equation.[S7]

$$\Phi_\Delta = \Phi_{ICG}\left(\frac{K_{sample}}{K_{ICG}}\right)\left(\frac{A_{ICG}}{A_{sample}}\right)$$

where $K_{sample}$ and $K_{ICG}$ are the decomposition rate constants of DPBF by BDP-6 and ICG, respectively. $A_{sample}$ and $A_{ICG}$ represent the integral areas of the optical absorption bands between 750 and 850 nm by BDP-6 and ICG, respectively.

**1.7 Photothermal Effect and Photothermal Conversion Efficiency Calculation**

For the purpose of evaluating the photothermal ability of BDP-6, we discussed the effect of power density and concentration on conversion efficiency. The temperature of solution was monitored by UT321 Digital Thermometers (UNI-T) at an interval of 30 s. Different concentrations (0 μM, 5 μM, 10 μM, 20 μM and 50 μM) of 1 mL BDP-6 aqueous solution were placed in quartz cuvette and irradiated by 808 nm laser at different power densities (0.5, 1.0 and 1.5 W $cm^{-2}$) for 10 min, respectively. The temperature changes were monitored during irradiation.

To evaluate the photothermal conversion efficiency of BDP-6, 1.0 mL of BDP-6 aqueous solution (50 μM) was irradiated with 808 nm irradiation at 1.5 W $cm^{-2}$ continually until the temperature elevation reached a steady state for 10 min, which naturally cooling down to the ambient temperature for 15 min afterwards. The temperature was measured every 30 s with a R500 series of InfraRed Camera (Avio, Japan). The water was used as a control. The photothermal conversion efficiency ($\eta$) was measured according to the reported method.[S8]

$$\eta = \frac{hs(T_{Max} - T_{Surr}) - Q_{Dis}}{I(1 - 10^{-A_{808}})}$$

$h$ is the heat transfer coefficient; $s$ is the surface area of the container. $Q_{Dis}$ represents heat that is dissipated from the laser mediated by the solvent and container. $I$ is the laser power and $A$ is the absorbance at 808 nm.

$$hs = \frac{mC_{Water}}{\tau_s}$$

$m$ is the mass of the solution containing the photoactive material, $C$ is the specific heat capacity of the solution ($C_{water}$ = 4.2 J/(g °C)), and $\tau_s$ is the associated time constant.

$$t = -\tau_s \ln(\theta)$$

$\theta$ is a dimensionless parameter, known as the driving force temperature.

$$\theta = \frac{T - T_{Surr}}{T_{Max} - T_{Surr}}$$

$T_{Max}$ and $T_{Surr}$ are the maximum steady state temperature and the environmental temperature, respectively.

### 1.8 Quantum-Chemical Calculations of BDP-6

The molecular geometries of BDP-6 were investigated by using the density functional theory (DFT) and time-dependent density functional theory (TDDFT) calculations. Becke's three-parameter hybrid exchange functions with Lee-Yang-Parr gradient-corrected correlation functional (B3-LYP functional) and the 6-31G (d, p) basis set are the most reasonable choice to be in good accordance with the experimental results. All quantum-chemical calculations were done with the Gaussian 09 suite. The vibrational frequencies of the optimized structures were calculated to ensure that the optimized structures of molecules have no imaginary vibrational frequencies. Electronic transition energies were obtained by the DFT method with the B3LYP functional and 6-31 G (d, p) basis set. The natural transition orbitals (NTOs) calculations were carried out to characterize the absorption transitions and the intersystem crossing between $S_1$ and $T_1$ state.

### 1.9 Minimum Inhibitory Concentration Assay of BDP-6

The minimum inhibitory concentration (MIC) is defined as the lowest concentration of antiseptic that inhibits the growth of an organism. The multidrug-resistant bacteria (MRSA) were cultured to $0.8\times10^8$ CFU mL$^{-1}$ in the Luria-Bertani (LB) medium, whose density was determined by the absorbance at the wavelength of 600 nm. Then, 7.5 μL of bacterial suspension was diluted by 3 mL of PBS to the density of $2\times10^5$ CFU mL$^{-1}$. The BDP-6 solution was prepared at the

concentration of 1024 μg mL$^{-1}$, and then a series of solutions were prepared by double dilution method. The BDP-6 at different concentrations (64~1024 μg mL$^{-1}$, 100 μL) were incubated with bacterial suspension (2×10$^{5}$ CFU mL$^{-1}$, 100 μL), which were irradiated by 808 nm NIR at a power density of 1.2 W cm$^{-2}$ laser for 10 min, and then 5 μL of bacterial suspension was moved from each well into the standard ager plate. The suspensions were diluted in 1:1000 with normal saline. The diluted suspensions (50 μL) were spread on a standard ager plate. These standard ager plates were incubated at 37 ℃ for 24 h. After incubation, the bacterial colonies were counted to evaluate the antibacterial property. Each group contained three duplicate samples.

### 1.10 Evaluation of *In Vitro* Photothermal and Antibacterial Performances

The antibacterial properties of PU and PU-GHB were determined by using *Staphylococcus aureus* (*S. aureus*), *Escherichia coli* (*E. coli*), methicillin-resistant *Staphylococcus aureus* (MRSA) and vancomycin-resistant *Enterococcus* (VRE). Four kinds of bacteria were respectively cultured to 0.8×10$^{8}$ CFU mL$^{-1}$. Then, 225 μL of bacteria suspension was centrifuged at 4000 rpm for 4 min. The collected bacteria were re-suspended by 9 mL of PBS to the density of 2×10$^{6}$ CFU mL$^{-1}$. The samples (PU, PU-GHB) which were sterilized by ultraviolet were added into 24-well plate with 500 μL of bacterial suspension. All samples were divided into the following three parts: 1) the control group with and without 808 nm NIR at a power density of 1.2 W cm$^{-2}$ laser for 10 min, 2) the PU group with and without irradiating, 3) the PU-GHB group with and without irradiating. Each group contained three duplicate samples. The bacteria suspension was diluted for 1000 times, and then the diluted suspensions (50 μL) were spread on a standard ager plate. These standard ager plates were incubated at 37 ℃ for 24 h. After incubation, the bacterial colonies were counted to evaluate the antibacterial property. To evaluate the synergistic antibacterial effects of the materials, the antibacterial activity of single thermal effect was assessed by the addition of ascorbic acid, and ROS was tested alone in ice-water bath.

Micromorphology observation of bacteria: SEM was used to observe the morphology of bacteria. After 808 nm NIR treatment or not, the bacteria in different

groups were centrifuged at 4000 rpm for 4 min and fixed with 2.5% glutaraldehyde for 24 h at 4 ℃. The fixed bacteria were sequentially dehydrated with increasing concentrations of ethanol (25%, 50%, 75%, 87.5%, 100%, 100% and 100%, each step was maintained for 5 min) in order, and then naturally dried to be observed by SEM.

Observation of bacteria in solution: CLSM was used to observe the bacteria. After 808 nm NIR treatment or not, the bacteria in different groups were stained with SYTO9 and propidium iodide for 10 min, and then were observed by CLSM with the usage of a 40× object lens.

Protein leakage assay: the BCA protein Assay Kit (Pierce®, Thermo Fisher, USA) was used for protein leakage assay. After 808 nm NIR treatment or not, the bacteria in different groups were centrifuged at 4000 rpm for 4 min and collected supernatant. The amount of the protein leakage was measured the absorbance at the wavelength of 562 nm by the microplate reader.

## 1.11 *In Vitro* Cell Viability Assay

Fibroblasts (L929) cells were selected to evaluate the biocompatibility. The L929 cells were cultured in Dulbecco's modified Eagle medium (DMEM) containing 10% of fetal bovine serum (FBS, Gibco, USA), 100 mg $mL^{-1}$ of streptomycin (Gibco, USA) and 100 U $mL^{-1}$ of penicillin (Gibco, USA) at 37 ℃ with 5% $CO_2$ humidified atmosphere. When growing to 80%, the cells were diluted to $1\times10^5$ $mL^{-1}$, and added 500 μL into the 24-well plate. After 24 h, the supernatant of the plate was replaced. The samples were sterilized by ultraviolet and added into 24-well plate. The Control (L+), PU (L+) and PU-GHB (L+) groups were irradiated by 808 nm NIR at a power density of 1.2 W $cm^{-2}$ laser for 10 min. And then, all groups were incubated in an incubator for 24 h and tested by MTT assay. The absorbance of each well was measured by the microplate reader at the wavelength of 570 nm. Each group contained five duplicate samples.

## 1.12 Hermia Animal Model Treatment

Sprague-Dawley (SD) rats (eight weeks old) were used to simulate ventral hernia infection in clinic setting. All of the animal experiments were performed in compliance with the guidelines issued by the Ethical Committee of the Chinese Academy of Sciences (CAS). The SD rats were divided into six groups, including control with (control R(B+, L-)) and without bacteria (control L(B-, L-)), PU with (PU R(B+, L+)) and without NIR irradiation (PU L(B+, L-)) and PU-GHB with (PU-GHB R(B+, L+)) and without NIR irradiation (PU-GHB L(B+, L-)). Each group contained three duplicate samples. The MRSA were cultured to $0.8\times10^{8}$ CFU $mL^{-1}$, and then 6.25 mL of bacteria solution was centrifuged at 4000 rpm for 4 min. The collected bacteria were re-suspended by 100 μL PBS to $5\times10^{9}$ CFU $mL^{-1}$. The abdomen of each rat was shaved and sterilized. 8±0.04 mm holes were made on left and right sides of the abdomen. 2 μL of bacterial suspension was dripped on each sample of PU and PU-GHB which were sterilized by ultraviolet. Samples containing bacteria were sutured to the surface of the defected muscle, and the skins were sutured. Considering the autoimmune response of the rats and the safety to the light, the light groups were irradiated by 808 nm NIR at a power density of 1.0 W $cm^{-2}$ laser for 15 min.

Three rats in each group were sacrificed at 1, 4 and 7 d and the wounds were to be observed. The tissues around the samples were harvested and mixed with normal saline at the ratio of 1:10. The suspensions were diluted in 1:1000 with normal saline. The diluted suspensions (50 μL) were spread on a standard ager plate and incubated at 37 ℃ for 24 h. The bacterial colonies were counted to evaluate the number of bacteria in the muscle. In order to measure the number of bacteria and cells that adhered to the surface, the samples were observed by CLSM. At the moment, the tissues around the wound were respectively fixed in snap-frozen or 4% paraformaldehyde for protein analysis and histological analysis. For the protein analysis, the tissues were shredded and weighted, and added into the RIPA buffer solution (900 μL buffer for every 100 mg of tissues). The tissues were dispersed by a vortex mixer totally, and centrifuged at 12000 rpm for 15 min. The supernatant was collected and tested by ELISA assay. The tissues in 4% paraformaldehyde were embedded in paraffin and stained with

hematoxylin and eosin (H&E) staining, Gram staining and immuno-histochemical analyses (TNF-α and IL-6).

### 1.13 Statistical Analysis

Each experiment contained at least three parallel samples and presented as mean ± standard deviation (mean ± SD). Origin (version 2019b, MA, USA) and GraphPad Prism (version 8.0.2, CA, USA) were used to process, analyze and graph the collected data. One-way ANOVA and *t*-test were combined for analyzing the significance. The levels of significance differences were marked as follows: “*” for $P < 0.05$, “**” for $P < 0.01$, and “***” for $P < 0.001$.

## 2. Supporting Figures

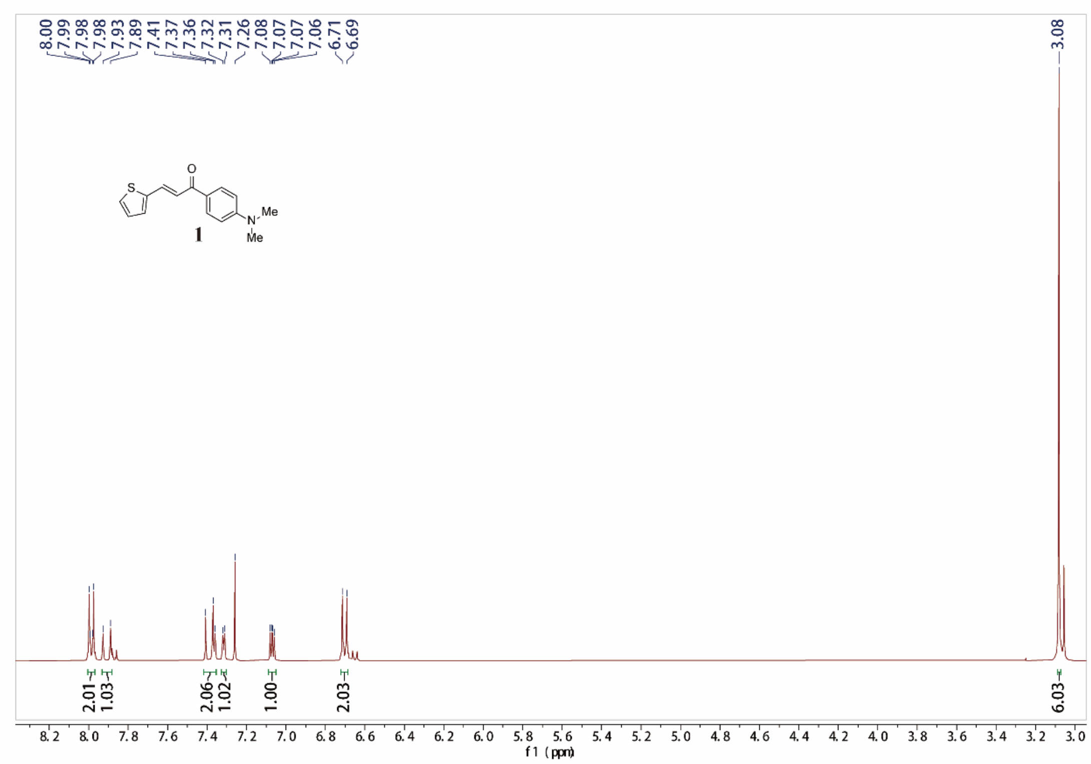


**Figure S1.** $^{1}$H-NMR spectrum of Compound 1 ($CDCl_3$, 400 MHz, 298 K).

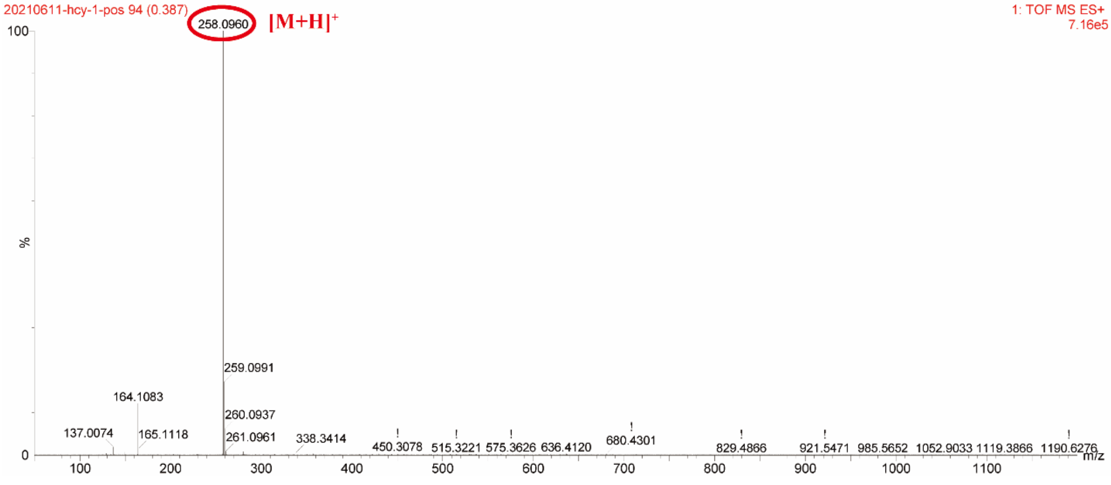


**Figure S2.** HRMS spectrum of Compound 1.

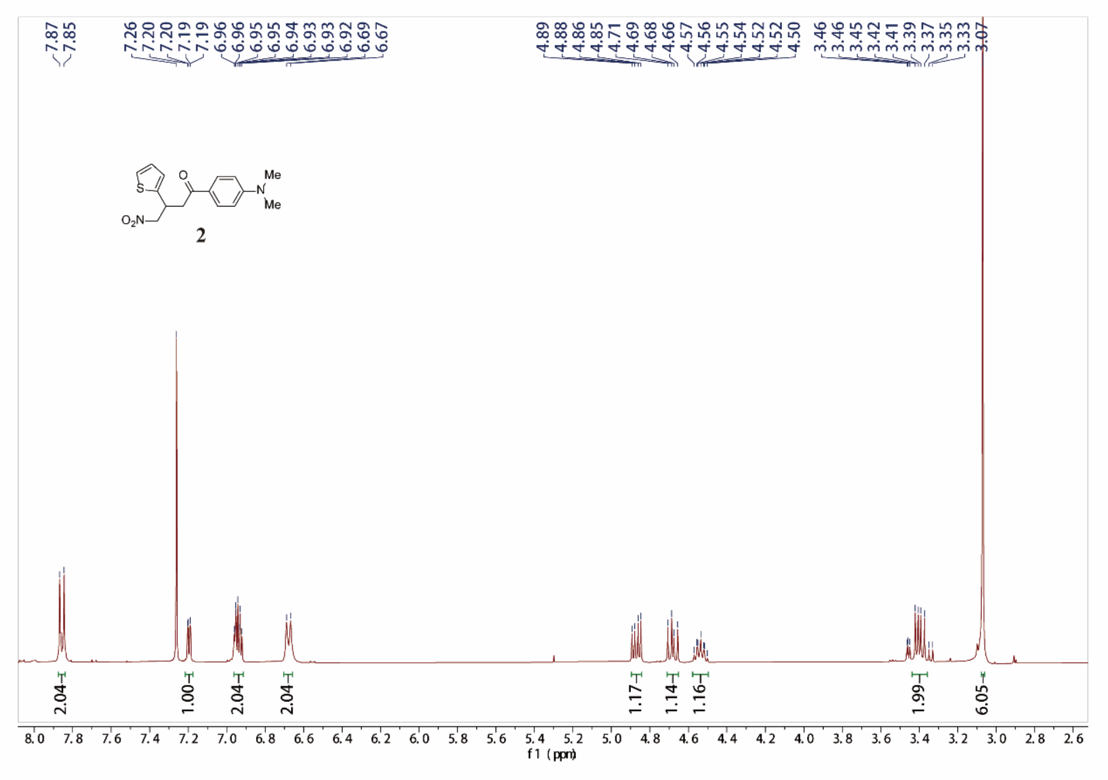

**Figure S3.** $^{1}$H-NMR spectrum of Compound 2 ($CDCl_3$, 400 MHz, 298 K).

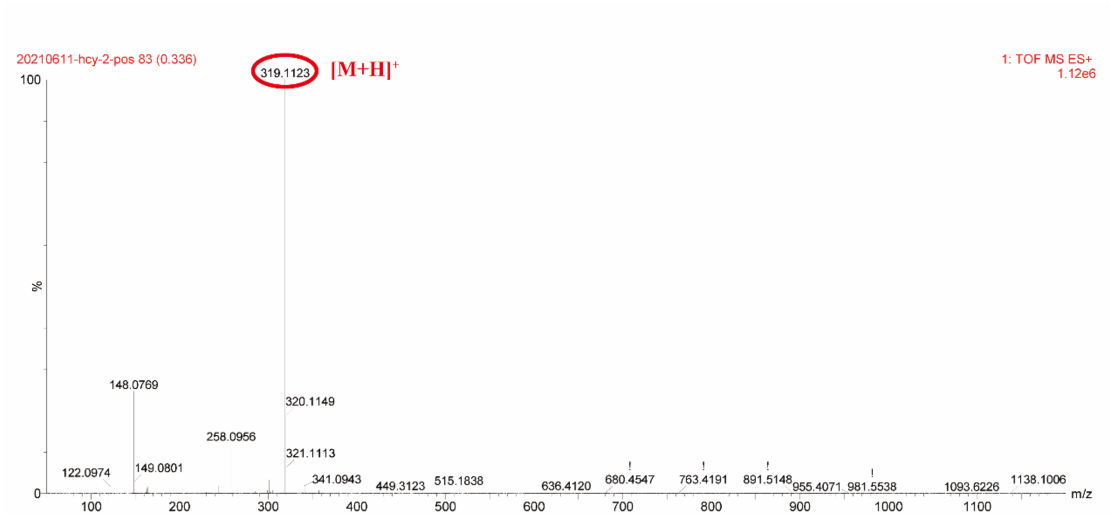

**Figure S4.** HRMS spectrum of Compound 2.

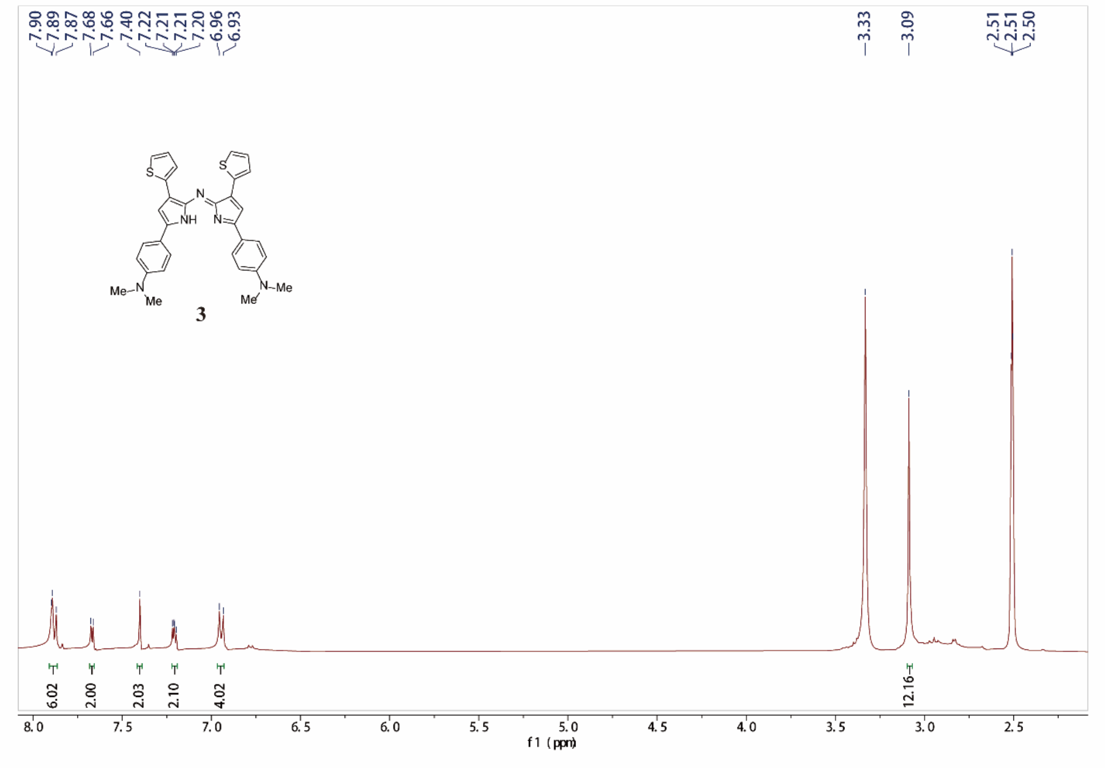


**Figure S5.** $^{1}$H-NMR spectrum of Compound 3 (DMSO-$d_6$, 400 MHz, 298 K).

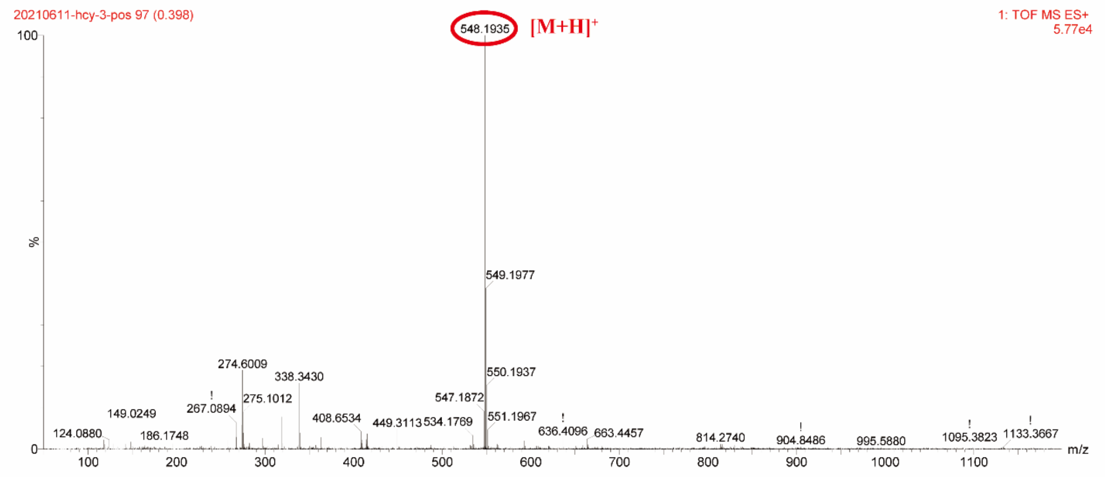


**Figure S6.** HRMS spectrum of Compound 3.

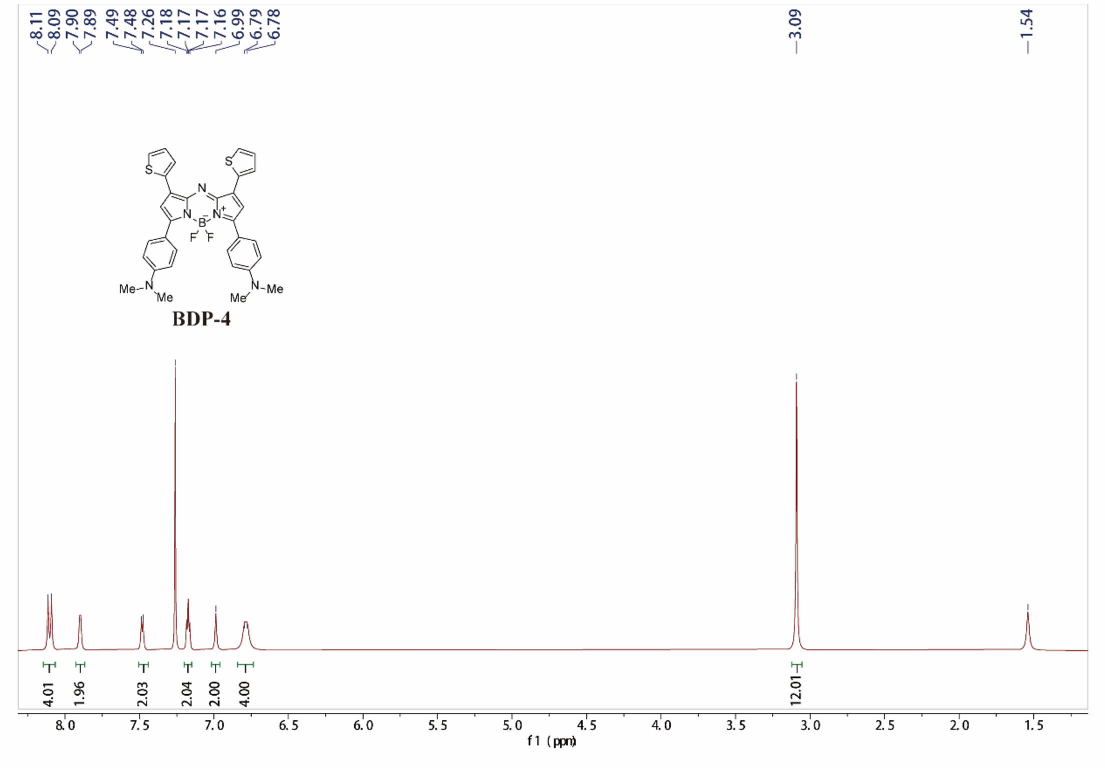


**Figure S7.** $^{1}$H-NMR spectrum of Compound BDP-4 ($CDCl_3$, 400 MHz, 298 K).

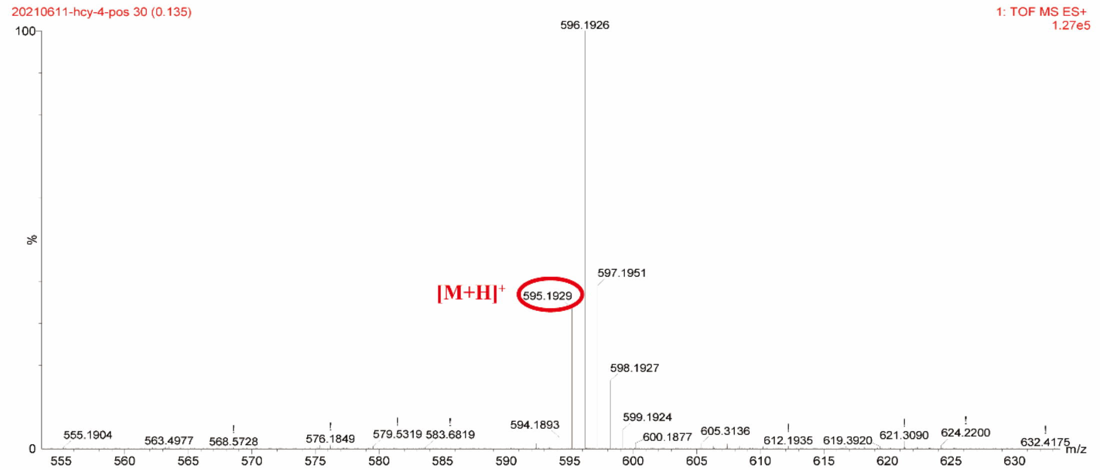


**Figure S8.** HRMS spectrum of Compound BDP-4.

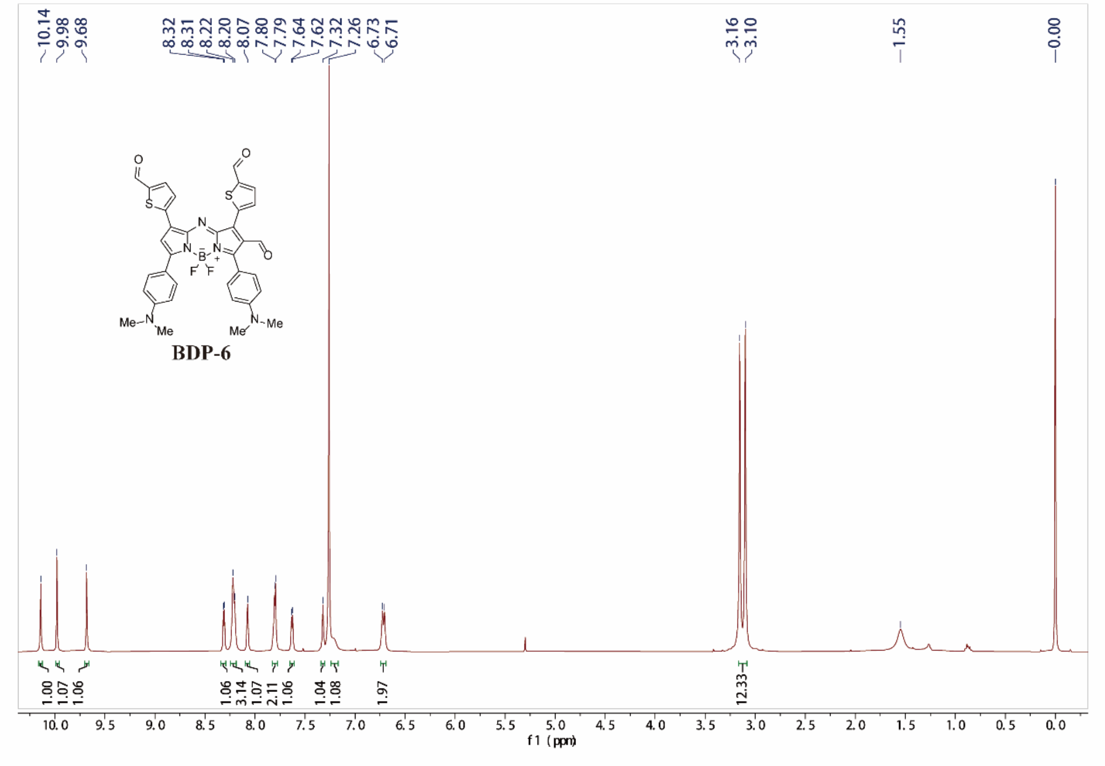

**Figure S9.** $^{1}$H-NMR spectrum of Compound BDP-6 ($CDCl_3$, 400 MHz, 298 K).

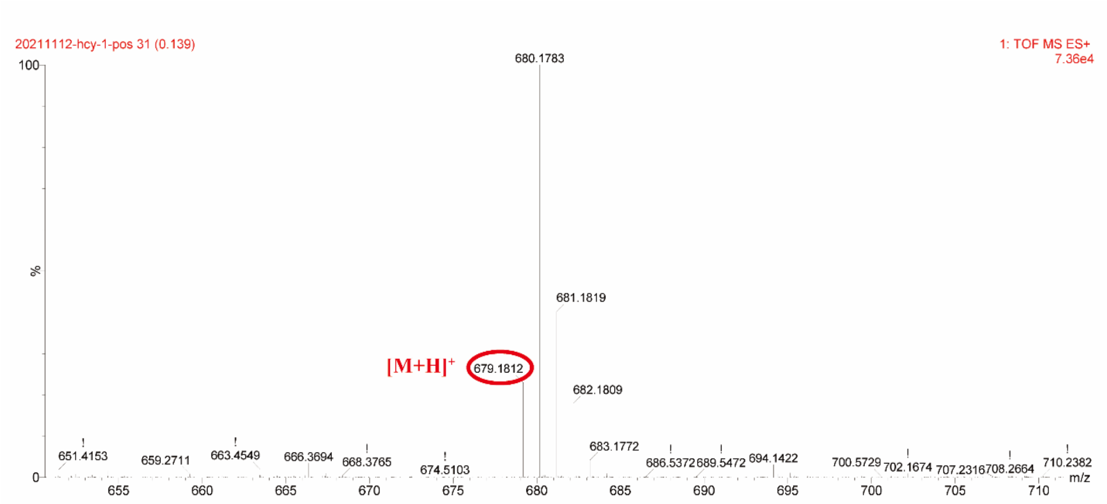


**Figure S10.** HRMS spectrum of Compound BDP-6.

**Table S1.** The optimized geometry and frontier molecular orbitals from DFT calculations of BDP-6 in the ground state ($S_0$).

| | $C_{35}H_{28}BF_2N_5O_3S_2$ | LUMO | HOMO |
|---|---|---|---|
| **BDP-6** | 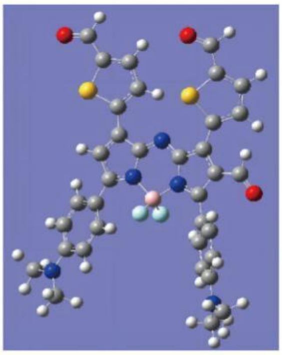 | 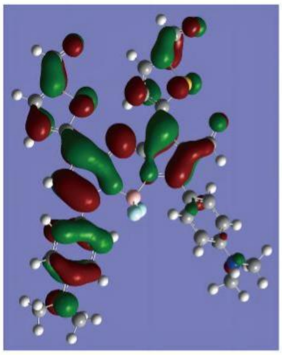 | 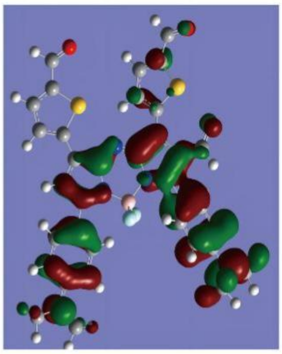 |
| $\triangle E_{gap}$ | 1.53 eV | | |
| $\triangle E_{S_1T_1}$ | 0.73 eV | | |

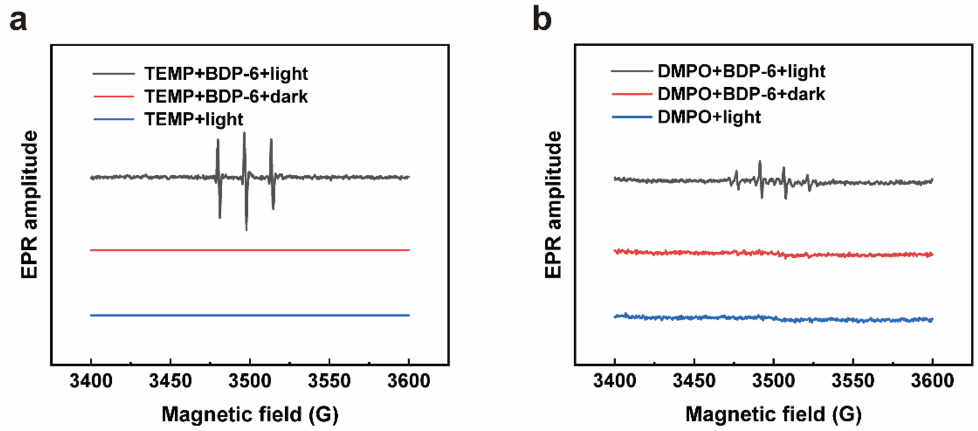


**Figure S11.** a) EPR spectra to detect $^1O_2$ generated BDP-6 under 808 nm light illumination, using TEMP as a spin trapper. The red line represents the EPR signal of the black group. b) EPR spectra to detect ·OH generated BDP-6 under 808 nm light illumination, using DMPO as a spin trapper.

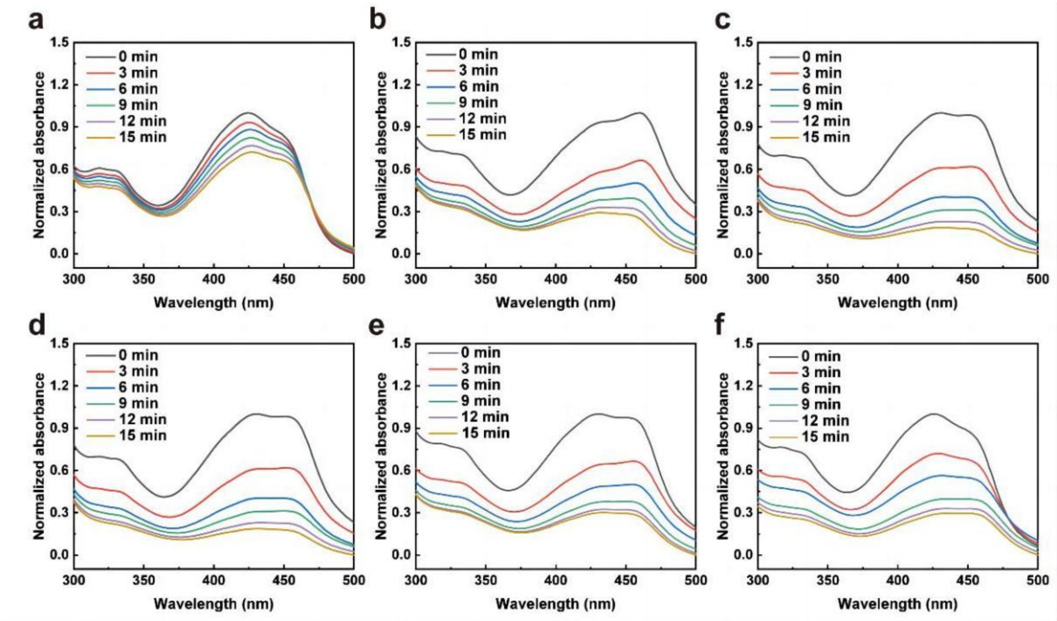


**Figure S12.** a), b) and c) UV-vis spectra of DPBF in the presence of BDP-6 (10 μM) aqueous solution upon 808 nm laser irradiation at different power densities (0.5, 1.0, 1.5 W $cm^{-2}$), respectively. The UV-vis spectra of DPBF in the presence of d) 10 μM BDP-6, e) 20 μM BDP-6 and f) 30 μM BDP-6 under the irradiation (at 1.5 W $cm^{-2}$) in water.

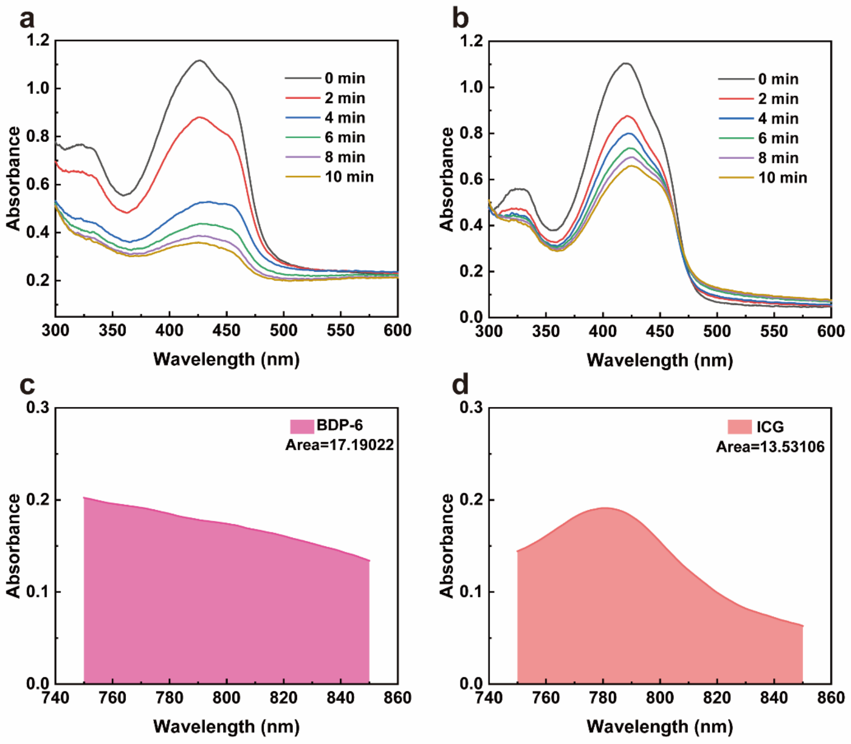


**Figure S13.** The absorption spectra of DPBF in the presence of a) BDP-6 and b) ICG upon 808 nm-light illumination at 1.5 W $cm^{-2}$, c) and d) represent the integral areas of the optical absorption bands between 750 and 850 nm by BDP-6 and ICG, respectively.

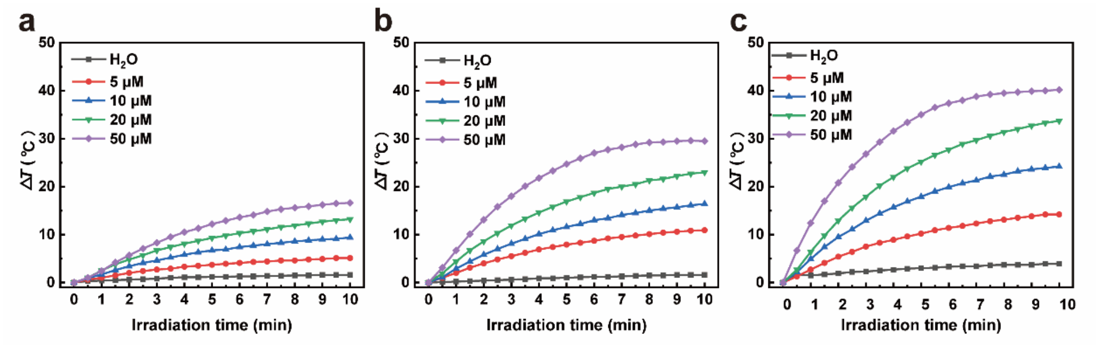


**Figure S14.** Temperature changes for aqueous solutions of BDP-6 with different concentrations (0, 5, 10, 20, 50 μM) upon 808 nm laser irradiation at a) 0.5 W cm$^{-2}$, b) 1.0 W cm$^{-2}$ and c) 1.5 W cm$^{-2}$, respectively.

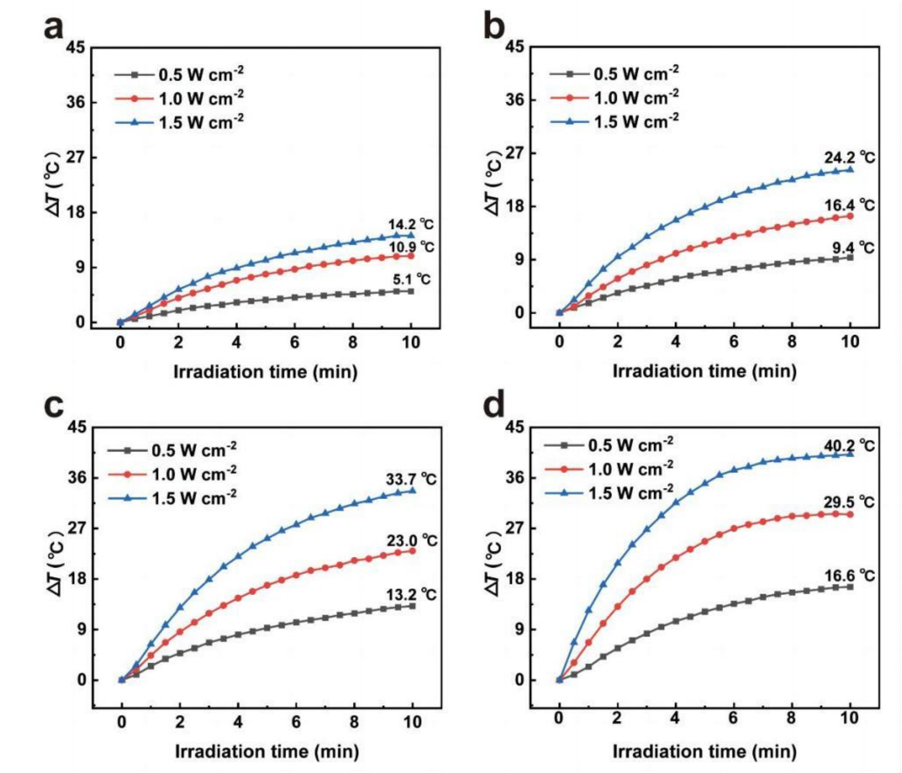


**Figure S15.** Photothermal heating of a) 5 μM BDP-6, b) 10 μM BDP-6, c) 20 μM BDP-6 and d) 50 μM BDP-6 in water upon 808 nm laser irradiation at different power densities (0.5, 1.0, 1.5 W cm$^{-2}$) for 10 min, respectively.

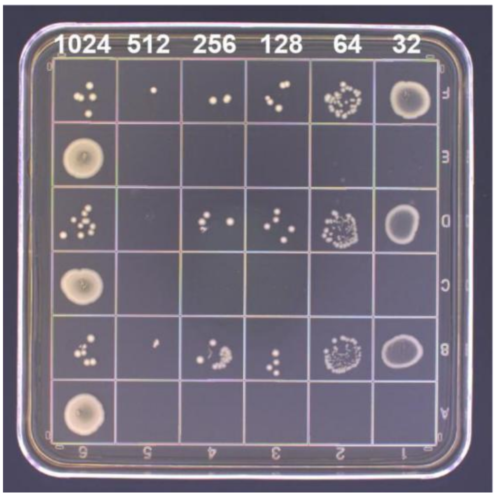


**Figure S16.** Minimum inhibitory concentration (MIC) of BDP-6 against MRSA.

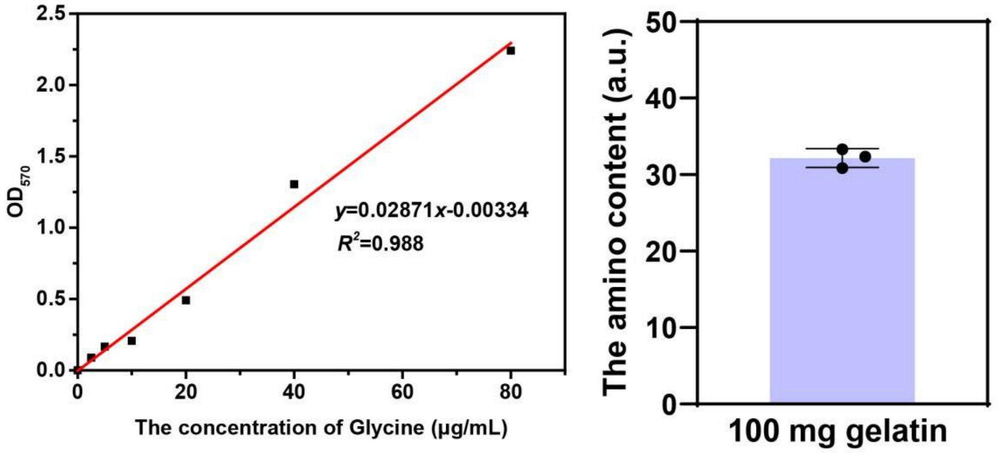


**Figure S17.** The amino content of the gelatin (100 mg), data are presented as means ± SD of *n*=3.

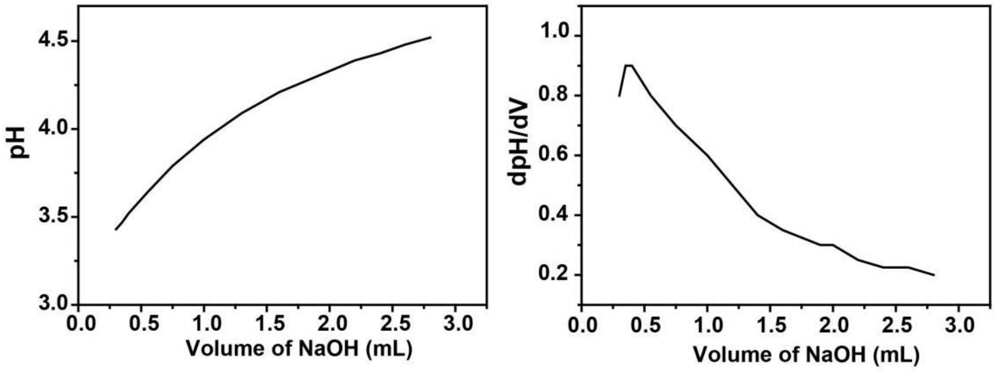


**Figure S18.** Potentiometric titration curves and its first differential curves of OHA.

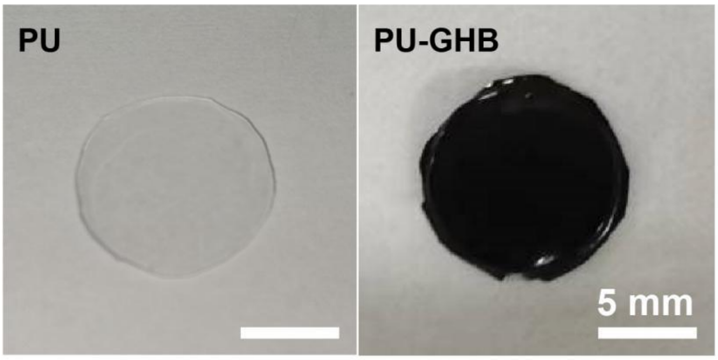


**Figure S19.** Photographs of PU and PU-GHB.

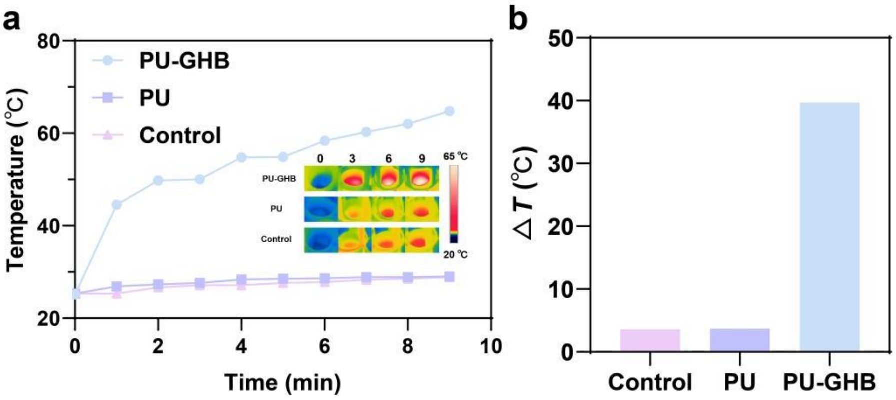


**Figure S20**. a) Heating curves and b) increased temperatures ($\Delta T$) of the samples after irradiation by 808 nm for 10 min.

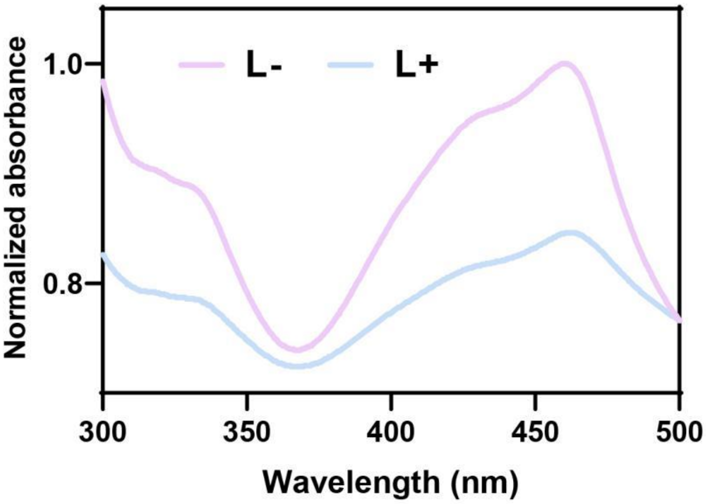


**Figure S21.** UV-vis spectra of DPBF for PU-GHB with (L+) and without (L-) NIR irradiation.

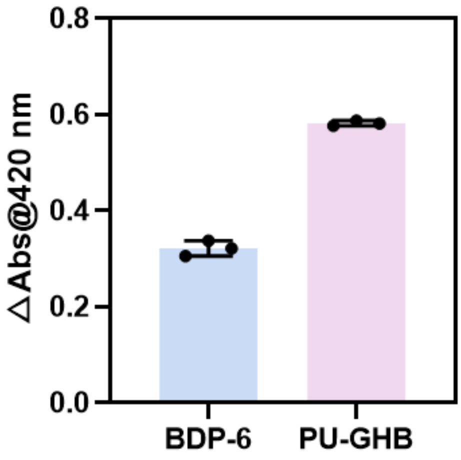


**Figure S22.** The photodynamic effect of free BDP-6 molecules at the equimolar concentration of the BDP-6 in PU-GHB, data are presented as means ± SD of *n*=3.

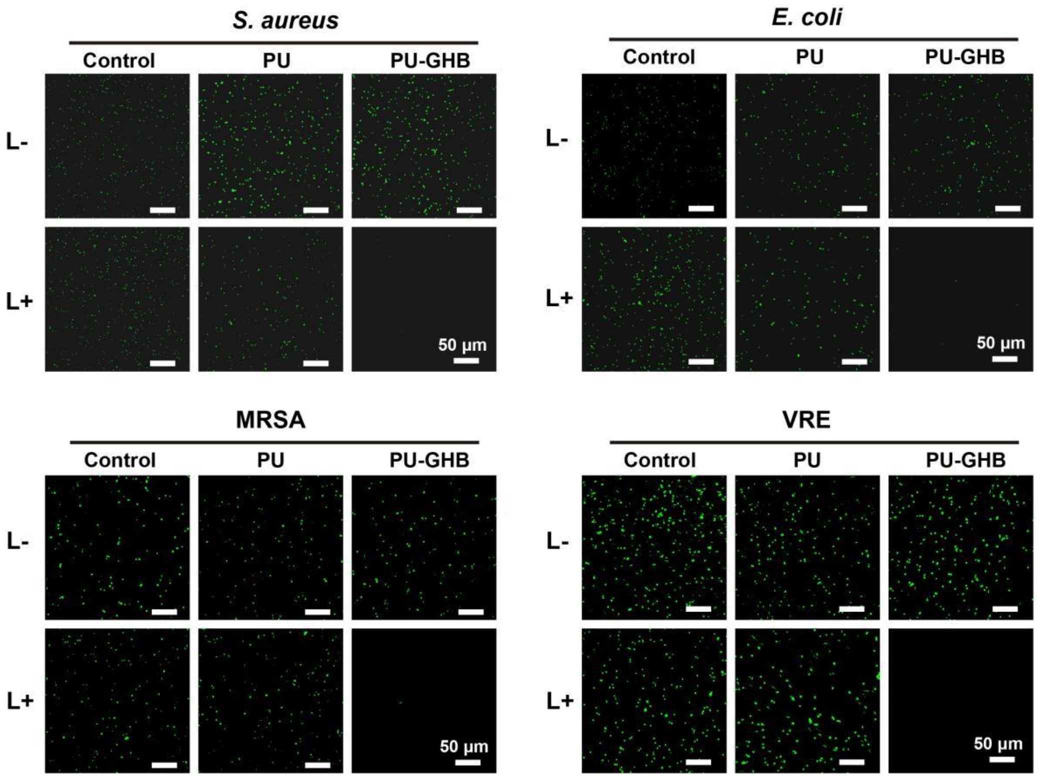


**Figure S23.** Representative CLSM images of planktonic *S. aureus*, *E. coli*, MRSA and VRE in the Control, PU and PU-GHB groups with (L+) and without (L-) NIR irradiation.

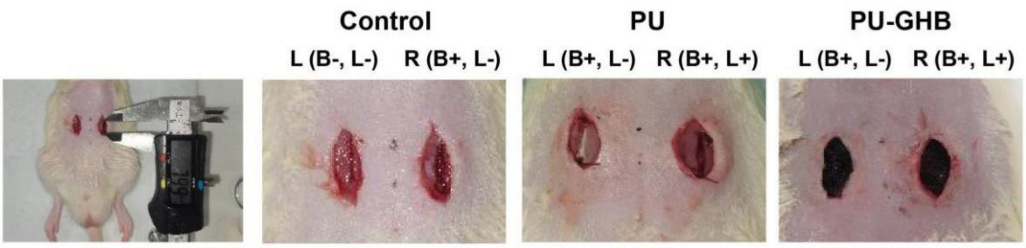


**Figure S24.** Photographs of implantation positions.

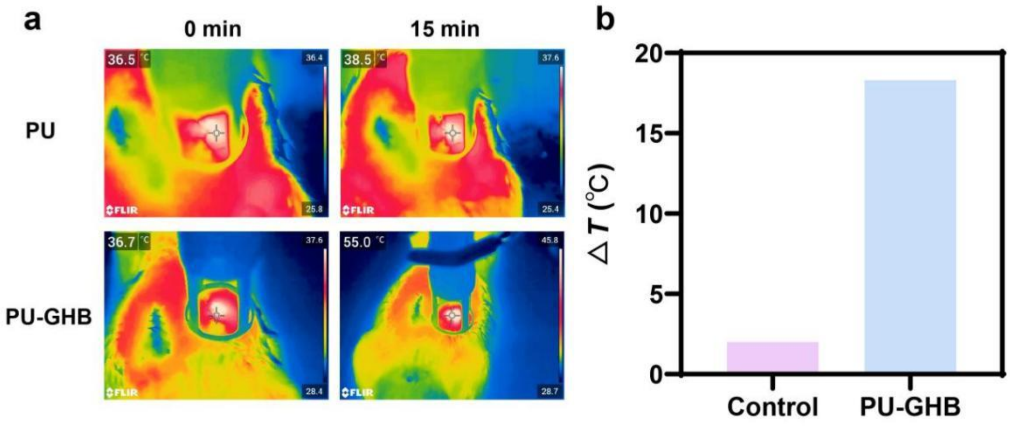


**Figure S25.** a) Infrared images of implantation positions. b) *In vivo* temperature increases of PU and PU-GHB after 808 nm NIR irradiation at a power density of 1.0 W $cm^{-2}$ for 15 min.

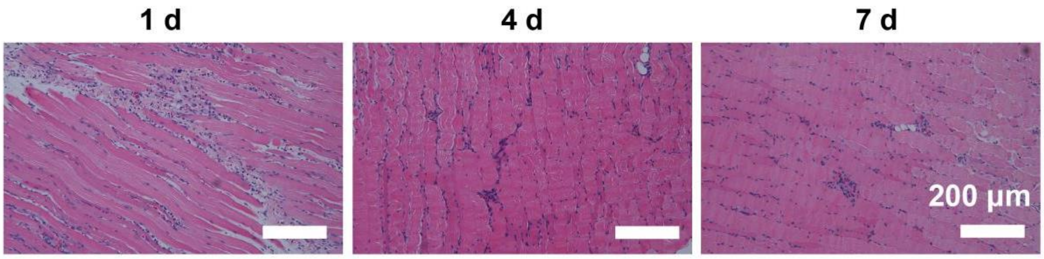


**Figure S26.** Histological images of the tissues around the samples without bacteria at 1, 4 and 7 d.

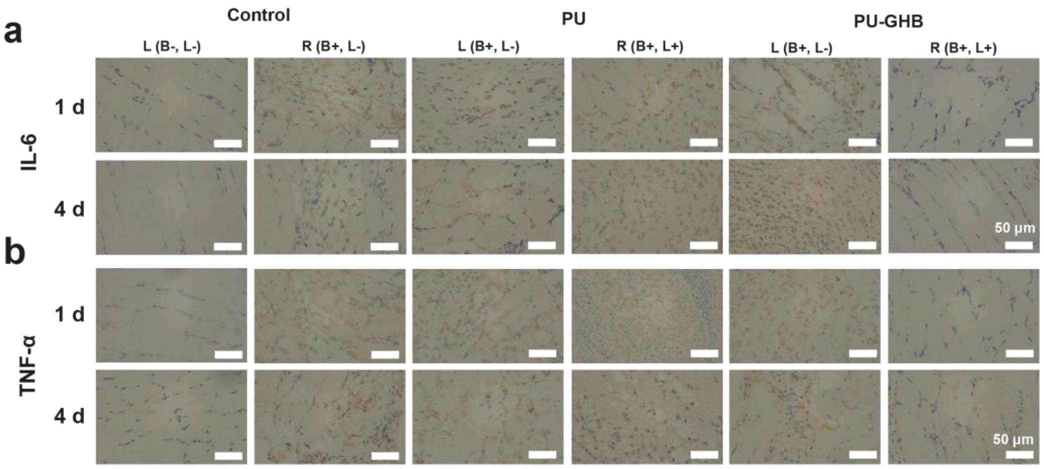


**Figure S27.** The immunohistochemical staining images of a) IL-6 and b) TNF-α at 1 d and 4 d after operation (the yellow color indicating positive areas).

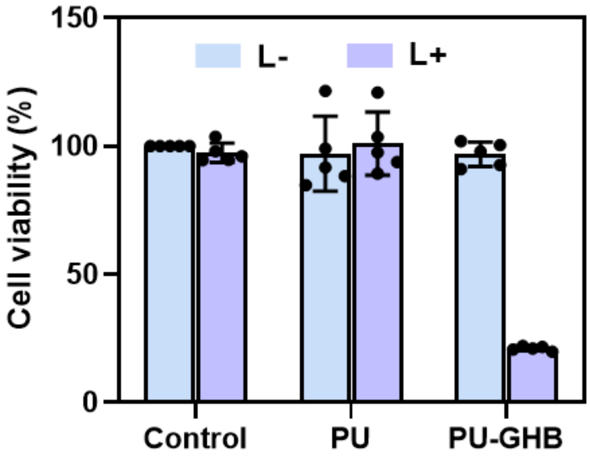


**Figure S28.** Cytotoxicity of PU, PU-GHB and control groups with (L+) and without (L-) NIR irradiation, data are presented as means ± SD of $n$=5.

**References**

[S1] L. Jiao, C. Yu, J. Li, Z. Wang, M. Wu, E. Hao, *J. Org. Chem.* **2009**, 74, 7525.
[S2] S. Kumar, H.B. Gobeze, T. Chatterjee, F. D'Souza, M. Ravikanth, *J. Phys. Chem. A* **2015**, 119, 8338.
[S3] Z. Emami, M. Ehsani, M. Zandi, R. Foudazi, *Carbohydr. Polym.* **2018**, 198, 509.
[S4] B. Liu, J. Jiao, W. Xu, M. Zhang, P. Cui, Z. Guo, Y. Deng, H. Chen, W. Sun, *Adv. Mater.* **2021**, 33, 2100795.
[S5] S. Wang, L. Shang, L. Li, Y. Yu, C. Chi, K. Wang, J. Zhang, R. Shi, H. Shen, G. I. N. Waterhouse, S. Liu, J. Tian, T. Zhang, H. Liu, *Adv. Mater.* **2016**, 28, 8379.
[S6] J. Zhang, F. Fang, B. Liu, J.-H. Tan, W.-C. Chen, Z. Zhu, Y. Yuan, Y. Wan, X. Cui, S. Li, Q.-X. Tong, J. Zhao, X.-M. Meng, C.-S. Lee, *ACS Appl. Mater. Interfaces* **2019**, 11, 41051.
[S7] L. Yang, B. Huang, S. Hu, Y. An, J. Sheng, Y. Li, Y. Wang, N. Gu, *Nano Res.* **2022**, 15, 4285.
[S8] D.K. Roper, W. Ahn, M. Hoepfner, *J. Phys. Chem. C* **2007**, 111, 3636.
